# Hippocampal sharp wave ripples underlie stress susceptibility in male mice

**DOI:** 10.1038/s41467-023-37736-x

**Published:** 2023-04-20

**Authors:** Nahoko Kuga, Ryota Nakayama, Shota Morikawa, Haruya Yagishita, Daichi Konno, Hiromi Shiozaki, Natsumi Honjoya, Yuji Ikegaya, Takuya Sasaki

**Affiliations:** 1 Laboratory of Chemical Pharmacology, Graduate School of Pharmaceutical Sciences, The University of Tokyo, 7-3-1 Hongo, Bunkyo-ku, Tokyo, 113-0033 Japan; 2 Department of Pharmacology, Graduate School of Pharmaceutical Sciences, Tohoku University, 6-3 Aramaki-Aoba, Aoba-Ku, Sendai, 980-8578 Japan; 3 Laboratory of Geriatric Medicine, Graduate School of Medicine, The University of Tokyo, 7-3-1 Hongo, Bunkyo-ku, Tokyo, 113-0033 Japan; 4 Center for Information and Neural Networks, 1-4 Yamadaoka, Suita City, Osaka, 565-0871 Japan; 5 Institute for AI and Beyond, The University of Tokyo, 7-3-1 Hongo, Bunkyo-ku, Tokyo, 113-0033 Japan

**Keywords:** Neural circuits, Stress and resilience, Molecular neuroscience

## Abstract

The ventral hippocampus (vHC) is a core brain region for emotional memory. Here, we examined how the vHC regulates stress susceptibility from the level of gene expression to neuronal population dynamics in male mice. Transcriptome analysis of samples from stress-naïve mice revealed that intrinsic calbindin (*Calb1*) expression in the vHC is associated with susceptibility to social defeat stress. Mice with *Calb1* gene knockdown in the vHC exhibited increased stress resilience and failed to show the increase in the poststress ventral hippocampal sharp wave ripple (SWR) rate. Poststress vHC SWRs triggered synchronous reactivation of stress memory-encoding neuronal ensembles and facilitated information transfer to the amygdala. Suppression of poststress vHC SWRs by real-time feedback stimulation or walking prevented social behavior deficits. Taken together, our results demonstrate that internal reactivation of memories of negative stressful episodes supported by ventral hippocampal SWRs serves as a crucial neurophysiological substrate for determining stress susceptibility.

## Abstract (web summary)

Stressful memories are a possible factor to induce psychiatric symptoms. Here, the authors demonstrate that stress susceptibility is related to memory consolidation mechanisms in the ventral hippocampus.

## Introduction

Psychiatric stress-induced symptoms, such as increased anxiety, decreased sociality, and depression, are not homogeneous but differ considerably across individuals, which are classified into stress susceptible and resilient phenotypes^1–3^. A number of studies in both humans and animals have revealed that these stress-induced responses arise from cooperative physiological activity of emotion-related brain regions, such as the medial prefrontal cortex (mPFC), amygdala (AMY), nucleus accumbens (NAc), and hippocampus^4–12^. In particular, the ventral hippocampus (vHC), which is distinct from the dorsal hippocampus (dHC)^13,14^, encodes affective and social memory^15–19^ and bidirectionally transmits contextual and aversive information to the mPFC and AMY^15,20–27^. As psychiatric stress responses are composed of affective, social, and contextual features, it is reasonable to speculate that the vHC is involved in the regulation of stress susceptibility, as shown by recent studies^5,16,28,29^. Interestingly, ventral hippocampal activity is enhanced, rather than attenuated, in stress susceptible mice^16,28–30^ and humans expressing depressive symptoms^31^. Considering the established role of the hippocampus in mnemonic functions, the processing of memories of stressful experiences in the vHC may be a primary factor in the development of stress-induced psychiatric symptoms. Thus, the aim of this study was to investigate whether and how stress susceptibility is triggered by memory processes in the vHC from the level of gene expression to neuronal population dynamics.

Here, we first screened stress susceptibility-related transcriptional targets in the vHC. Early studies identified stress-induced molecular factors^32–34^ and transcriptional networks, including key module hub genes related to stress susceptibility in emotion-related brain regions^2,29,35^. However, the molecular mechanisms that directly underlie the relationship between memory-related neuronal activity in the vHC and stress susceptibility remain to be elucidated. In addition, early studies quantified protein and gene expression in tissue samples collected from animals after stress exposure^2,29,34,35^. In this study, to exclude transcriptional changes caused by stress-induced pathological effects, we collected tissue samples from the vHC of stress-naïve mice before exposure to social defeat (SD) stress^36^. After identifying susceptibility-related genes and exploiting an a priori viral-mediated gene knockdown approach that enhances the stress resilience of mice, we examined the effect of this manipulation on ventral hippocampal neuronal activity using multiunit spike recordings. We found that poststress sharp wave ripples (SWRs) in the vHC, which trigger synchronous reactivation of stress memory-encoding neuronal ensembles and effective ventral hippocampal-amygdalar information transfer, exert pro-susceptibility effects. The causal role of this ventral hippocampal SWR-mediated memory reactivation in the expression of stress susceptibility was tested by an online feedback manipulation technique.

## Results

### Calbindin expression in the vHC is associated with stress susceptibility

**Fig. 1:**
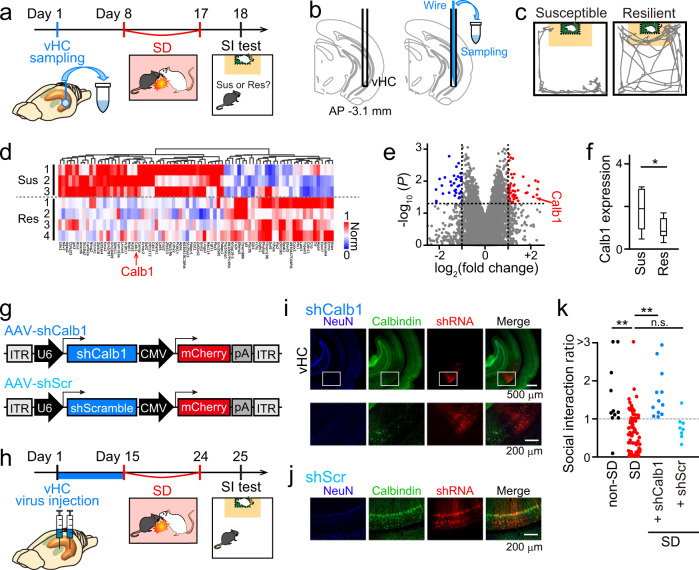
Calb1 expression in the vHC regulates stress susceptibility. **a** Experimental timeline of sample collection, chronic SD exposure, and the SI test. **b** Schematic illustration of sample collection from the vHC. A guide cannula was implanted into the vHC (left), and a metal wire (cyan) was then inserted through the cannula to collect a tissue sample (right). **c** Trajectories of representative susceptible (left) and resilient (right) mice in the SI test. The orange regions represent the interaction zone. **d** Heatmap showing hierarchical clustering of gene expression patterns collected from the vHC before SD stress. After SD stress exposure, three stress susceptible and four stress resilient mice were identified in subsequent SI tests and their gene expression patterns were compared. The 80 genes that showed a more than 2-fold difference in expression with a *P* value < 0.05 (corresponding to **e**) between the two groups are shown. **e** Volcano plot showing the fold change in the expression of and *P* value of individual genes in stress susceptible mice compared with stress resilient mice. The vertical dotted lines represent a 2-fold difference, and the horizontal dotted line represents the significance cutoff (one-sided Student’s *t*-test, *P*  =  0.05). The red and blue dots indicate significantly upregulated and downregulated genes, respectively, in stress-susceptible mice. **f** qPCR analysis of the expression levels of Calb1 (*n* = 13 and 9 mice). Box plots show center line as median, box limits as upper and lower quartiles, whiskers as minimum to maximum values. *Z* = 2.00, *P = 0.045, two-sided Mann–Whitney *U* test. **g** Structures of the AAV constructs. **h** Experimental timeline of virus injection. **i** Representative images showing the expression of Calb1 (green) and mCherry (shRNA, red) in the vHC in AAV-shCalb1-injected mice. The white boxes in the top panels are magnified in the bottom panels and show knockdown of Calb1 in shRNA-expressing ventral hippocampal neurons. **j** Same as i but for AAV-shScr-injected mice, showing highly overlapping expression of Calb1 and shRNA. **k** SI ratios of non-SD, SD mice, and SD mice injected with AAV-shCalb1 and AAV-shScr into the vHC (*n* = 12, 73, 12, and 8 mice). Each dot represents one mouse. Non-SD versus SD, *Z* = 3.73, **P = 5.7 × 10^−4^; SD versus AAV-shCalb1, *Z* = 4.23, **P = 2.3 × 10^−5^; SD versus AAV-shScr, *Z* = 0.82, *P* > 0.99, two-sided Mann–Whitney *U* test followed by Bonferroni correction.

First, we identified the transcriptional network in the vHC related to stress susceptibility (Fig. 1a). A small piece (~0.01 mm^3^) of tissue^36^ was microdissected from the left vHC of stress-naïve C57BL/6J mice for subsequent microarray analysis (Fig. 1b). The mice were allowed to recover from the surgery for one week and then subjected to a 10-min SD stress protocol in which they were exposed to an aggressive CD-1 mouse^1,37^ (termed SD mice). For mice that underwent vHC tissue sample collection, SD stress was applied every 2 days for 10 days to reduce postsurgery physical damage to the SD mice. The next day, the social behavior of the mice was assessed using a social interaction (SI) test. For each mouse, the SI ratio was calculated as the ratio of occupancy time in the interaction zone in the target session to that in the no-target session. SD mice that had an SI ratio of less than and more than 1 were identified as susceptible and resilient individuals, respectively (Fig. 1c). As a first screening step, we applied transcriptome analysis to compare gene expression patterns in the tissue of seven mice that were collected before SD stress. After applying SD stress, of the seven mice, three mice and four mice showed stress susceptible and resilient types, respectively, in subsequent SI tests. Differentially expressed genes between these susceptible and resilient mice were identified with a false discovery rate-adjusted p-value cutoff of 0.05 (Fig. 1d, pseudocolor image), which were classified based on gene ontology (GO) analysis (Fig. 1d, dendrogram). In total, 48 and 32 genes were highly expressed in susceptible mice and resilient mice, respectively (Fig. 1e). Representative genes showing greater expression in the susceptible mice than in the resilient mice included calcium signaling-related proteins such as calbindin (*Calb1*), calneuron 1 (*Caln1*), and calcium/calmodulin-dependent protein kinase II alpha (*Camk2a*). In the phenotype-specific transcriptional network, we focused on *Calb1* expression as a key factor, as the calbindin-D28K (abbreviated Calb1) protein modulates the dynamics of intracellular calcium and has been reported to be critically involved in hippocampal memory functions^38,39^. Quantitative PCR (qPCR) analysis from different sets of the ventral hippocampal tissue samples further confirmed that *Calb1* expression levels were enhanced in susceptible mice compared with resilient mice (Fig. 1f; *n* = 13 and 9 susceptible and resilient mice, respectively; *Z* = 2.00, *P* = 0.045, Mann–Whitney *U* test). These results suggest that manipulation of *Calb1* expression has the potential to regulate pro-susceptibility memory mechanisms and that Calb1 may be an attractive target for increasing stress resilience.

To address this idea, we induced vHC-specific knockdown of Calb1 by bilateral injection of a viral vector expressing an shRNA targeting *Calb1* mRNA (AAVdj-shCalb1-mCherry; abbreviated AAV-shCalb1) into the vHC (Fig. 1g, h). As a control for the effects of viral transfection, a nontargeting shRNA-expressing vector (AAVdj-shScramble-mCherry; abbreviated AAV-shScr) was injected into the vHC. Immunostaining of tissue from AAV-shCalb1-injected mice confirmed that viral-mediated expression of shCalb1 caused a marked reduction in the Calb1 protein level in mCherry-expressing neurons in the vHC (Fig. 1i), while Calb1 protein levels remained normal in mCherry-expressing ventral hippocampal neurons in AAV-shScr-injected mice (Fig. 1j). Two weeks after virus injection, these mice were subjected to chronic SD stress for consecutive 10 days, similar to a general protocol of SD stress, and a subsequent SI test. In our protocol, 78.1% (57 out of 73) and 21.9% (16 out of 73) of SD mice had an SI ratio of less than and more than 1, respectively, and were thus identified as susceptible and resilient individuals, respectively (Fig. 1k). The SI ratios of the SD mice were significantly lower than those of the non-SD mice (Fig. 1k; *n* = 12 mice, *Z* = 3.73, *P* = 5.7 × 10^−4^, Mann–Whitney *U* test followed by Bonferroni correction). In this condition, 12 SD mice injected with AAV-shCalb1 had SI ratios that were significantly higher than those of the SD mice (Fig. 1k; *n* = 12 mice, *Z* = 4.23, *P* = 2.3 × 10^−5^, Mann–Whitney *U* test followed by Bonferroni correction). AAV-shScr injection did not show such a significant effect in SD mice (Fig. 1k; *n* = 8 mice, *Z* = 0.82, *P* > 0.99, Mann–Whitney *U* test followed by Bonferroni correction). The same behavioral analysis was applied to mice injected with AAVdj-Calb1^OE^ in which Calb1 was overexpressed in ventral hippocampal neurons in a virus-infected area (Supplementary Fig. 1a–c). However, these mice did not show significant differences in SI ratios, compared with the SD mice (Supplementary Fig. 1d; *n* = 8 mice), demonstrating that the overexpression of Calb1 has no prominent effects on stress-induced social interaction deficits. Taken together, downregulation of *Calb1* expression in the vHC specifically increases resilience to SD stress, highlighting the contribution of Calb1 in the vHC in the expression of a pro-susceptibility phenotype. In the following analyses, we exploited vHC-specific Calb1 knockdown to evaluate the neurophysiological mechanisms that may explain differences in stress resilience.

### Poststress Calb1-dependent ventral hippocampal SWRs are associated with stress susceptibility

**Fig. 2:**
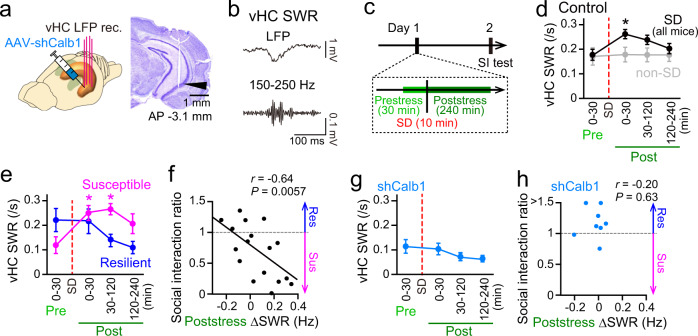
Poststress ventral hippocampal SWRs differ between stress-susceptible and stress-resilient mice. **a** (Left) LFP recordings were obtained from the vHC after injection of AAV-shCalb1. (Right) Histological confirmation of a recording site in the vHC CA1 cell layer in a cresyl-stained section. The arrowhead indicates the track of the electrode. The same results were obtained from 72 mice with electrodes implanted in the vHC. **b** Unfiltered and bandpass-filtered (150–250 Hz) LFP traces of a representative ventral hippocampal SWR. **c** Schematic illustration of the recording time course. The difference in poststress neuronal activity between the prestress and 0–2 h poststress period was computed on day 1, and several mice were subjected to an SI test on day 2. **d** Changes in the rates of ventral hippocampal SWRs in SD mice (black, *n* = 23 mice). For comparison, the same recordings were performed for non-SD mice (gray, *n* = 6 mice). 0–30-min pre versus 0–30-min post periods, *Z* = 2.65, *P = 0.024, two-sided Mann–Whitney *U* test followed by Bonferroni correction. The data are the mean ± SEM. **e** The data from the SD mice shown in d were plotted separately for mice that showed stress susceptibility (magenta, *n* = 12 mice) and resilience (blue, *n* = 5 mice), as defined by a subsequent SI test. 0–30-min pre versus 0–30-min post periods, *Z* = 3.02, *P = 0.0075; 0–30-min pre versus 30–120-min post periods, *Z* = 3.15, *P = 0.0049, two-sided Mann–Whitney *U* test followed by Bonferroni correction. The data are the mean ± SEM. **f** Relationship between the poststress ΔSWR rate and SI ratio. Each dot represents a mouse (*n* = 17 mice). *R* = –0.64, *P* = 0.0057. **g** Same as d but for mice injected with AAV-shCalb1 into the vHC (*n* = 8 mice). *P* > 0.05 in all post periods versus the 0–30-min prestress period, two-sided Mann–Whitney *U* test followed by Bonferroni correction. The data are the mean ± SEM. **h** Same as f but for mice injected with AAV-shCalb1. No significant Pearson’s correlation (*P* = 0.63, two-sided) was found between the poststress ΔSWR rate and SI ratio.

To examine how Calb1 expression alters neuronal activity patterns in the vHC, we obtained electrophysiological recordings in the vHC after injection of AAV-shCalb1 using tetrode arrays (Fig. 2a). First, we compared overall ventral hippocampal local field potential (LFP) power at the delta (1–4 Hz), theta (6–10 Hz), slow-gamma (20–50 Hz), and fast-gamma (60–100 Hz) bands during quiescent periods in a home cage among control (no virus injection; *n* = 23 mice), AAV-shCalb1-injected (*n* = 11 mice), and AAV-shScr-injected mice (*n* = 5 mice). No significant differences in the power at these frequency bands were found among these mouse groups (Supplementary Fig. 2a; Mann–Whitney U test, *P*  >  0.05). The same non-significant results were observed when the statistical analyses were applied to all frequency bands ranging from 1 to 100 Hz (Supplementary Fig. 2b; Mann–Whitney *U* test, *q*  >  0.05, FDR corrected). From the same LFP traces, we next detected SWRs consisting of short-lasting (~100 ms) and large-amplitude sharp-wave and fast oscillatory ripples (150–250 Hz) (Fig. 2b). In the home cage, no significant difference in the rate of ventral hippocampal SWRs was found among these three mouse groups (Supplementary Fig. 2c; *P* > 0.05, Mann–Whitney *U* test). In addition, the same recordings and analyses were performed from mice that experienced a novel open field for 10 min to investigate whether a novel experience alone without SD stress affects these LFP signals (Supplementary Fig. 2d–f). No significant differences in delta, theta, slow-gamma, and fast-gamma power, and the rate of SWRs in the vHC were observed between control and AAV-shCalb1-injected mice (Mann–Whitney *U* test, *P* > 0.05 in all analyses). These results suggest that changes in Calb1 expression do not strongly affect neuronal population activity in the vHC under stress-naïve conditions.

We next assessed whether Calb1 expression affects SWRs in mice after exposure to SD stress (Fig. 2b), which is considered to play crucial roles in the consolidation and stabilization of memory related to experiences^40–42^. For each SD mouse, a recording session included a 60-min prestress period in the home cage, a 10-min SD period involving the same protocol as stated above, and a 240-min poststress period in the same home cage without aggressors (Fig. 2c). In control mice, ventral hippocampal SWRs were significantly more frequent for up to 30 min in the poststress period than in the prestress period (Fig. 2d, black; *n* = 23 mice, *P* < 0.05, Mann–Whitney *U* test followed by Bonferroni correction). No significant difference in the ventral hippocampal SWR rate was observed in the non-SD mice (Fig. 2d, gray; *n* = 6 mice, *P* > 0.05 for all periods). The same analysis was performed for SWRs in the dHC (Supplementary Fig. 3a). The SD-induced increase in the SWR rate was not observed in the dHC (Supplementary Fig. 3b; *P* > 0.05 for all periods). Simultaneous recordings of the dHC and vHC revealed that subsets of SWRs in these two subregions occurred independently after SD exposure (Supplementary Fig. 3c–f)^43,44^.

To further confirm this idea, we classified the SD mice shown in Fig. 2d into susceptible and resilient types based on the SI ratio obtained from a subsequent SI test the next day. Here, all of the SI tests were performed in the same room and same location and with the same experimental conditions so that the SD mice more strongly recalled an episode of SD stress. In addition, the physical load due to device implantation might induce stronger stress-induced effects. Taken together with these effects, we verified that these experimental conditions were sufficient to yield both susceptible and resilient mouse types (Supplementary Fig. 4). We separately computed the ventral hippocampal SWR rate in these mice and found that the susceptible mice specifically showed a significant increase in the ventral hippocampal SWR rate for up to 2 h in the poststress period (Fig. 2e; susceptible, *n* = 12 mice, *P* < 0.05, Mann–Whitney *U* test followed by Bonferroni correction), whereas the resilient mice did not show such a significant change (Fig. 2e; resilient, *n* = 5 mice, *P* > 0.05, Mann–Whitney *U* test followed by Bonferroni correction). Plotting the relationship between the difference in the ventral hippocampal SWR rate between the prestress period and the 0–2-h poststress period (poststress ΔSWR) and the SI ratio computed from the subsequent SI test revealed a significant negative correlation (Fig. 2f; *n* = 17 mice, *r* = –0.64, *P* = 0.0057). The same recordings and analyses were applied to AAV-shCalb1-injected mice. Overall, AAV-shCalb1-injected mice showed no significant changes in the ventral hippocampal SWR rate during the poststress period (Fig. 2g; *n* = 11 mice, *P* > 0.05 for all periods, Mann–Whitney *U* test followed by Bonferroni correction) and showed significantly higher SI ratios, compared with the non-injected mice under the same experimental conditions as shown in Fig. 2f (Fig. 2h; *n* = 8 mice, *Z* = 2.53, *P* = 0.011, Mann–Whitney *U* test). No significant correlation was found between poststress changes in the ventral hippocampal SWR rates and SI ratios in these AAV-shCalb1-injected mice (Fig. 2h; *n* = 8 mice, *r* = –0.20, *P* = 0.63). On the other hand, AAV-shScr-injected mice, similar to the SD mice, showed significant increases in the ventral hippocampal SWR rate during the poststress period (Supplementary Fig. 2g; *n* = 5 mice). These results demonstrate that the reduction of Calb1 expression in the vHC abolishes the stress-induced increase in the ventral hippocampal SWR rate and subsequent social interaction deficits.

### SWR-induced reactivation of SD-encoding ventral hippocampal neurons

**Fig. 3:**
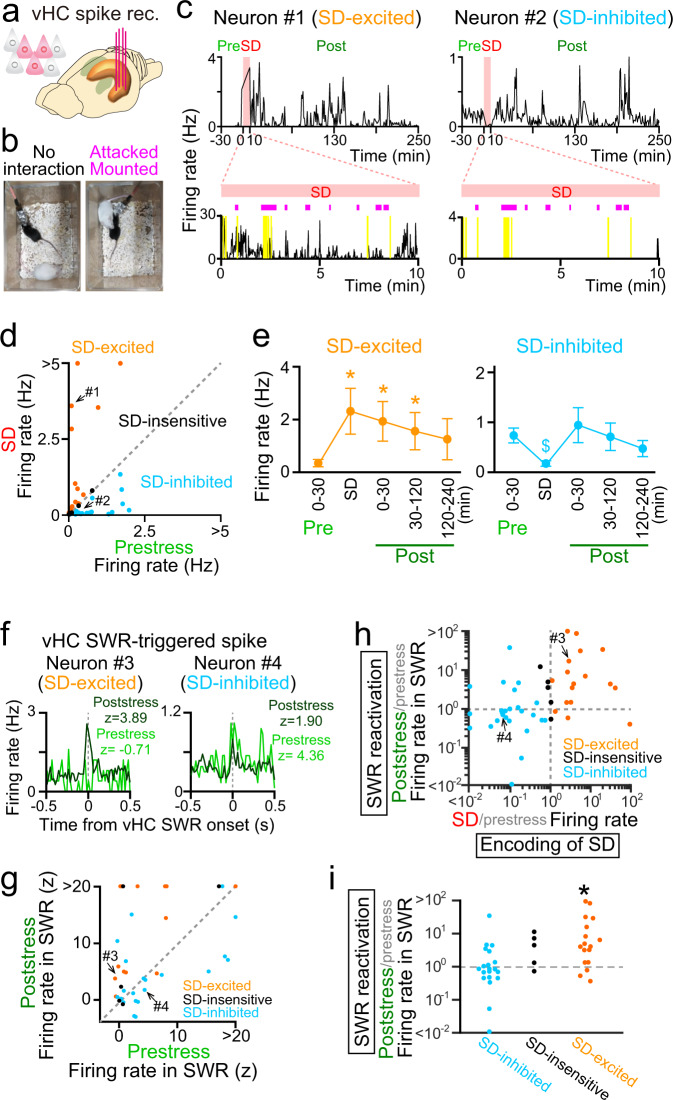
Reactivation of SD-encoding ventral hippocampal neurons by ventral hippocampal SWRs. **a** (Left) Multiunit spikes were recorded from ventral hippocampal CA1 neurons. **b** Images of the recording processes during SD, including during no interaction (left) and attacking or mounting (right). **c** (Top) Changes in instantaneous the firing rates (bin = 1 and 10 min in the rest and SD periods, respectively) of two representative vHC neurons showing significant increases (left, SD excited) and decreases (right, SD inhibited) in their firing rates during SD stress. (Bottom) The SD periods are magnified from the top panels (bin = 1 s). The magenta bars indicate periods of attacking or mounting of the SD mice. The yellow regions indicate periods that were removed from the analysis due to severe noise. **d** Scatterplot comparing neuronal firing rates between the prestress (2 h) and SD periods (*n* = 40 cells), shown in different colors depending on their relation to SD. Each dot represents a neuron. **e** Changes in the average firing rates of all SD-excited (*n* = 15) and SD-inhibited (*n* = 20) putative pyramidal neurons of each cell type. * and $ represent significant increases and decreases in rates, respectively, compared with the 30-min prestress period (SD-excited: versus SD, *P = 2.4 × 10^−4^; versus 0–30-min post, *P = 4.8 × 10^−4^; versus 30–120-min post, *P = 2.4 × 10^−4^; SD-inhibited: versus SD, ^$^*P* = 3.5 × 10^−4^, two-sided Wilcoxon signed rank test followed by Bonferroni correction). The data are the mean ± SEM. **f** Comparison of ventral hippocampal SWR-triggered spike rate changes between the prestress and poststress periods for representative SD-excited (left) and SD-inhibited (right) ventral hippocampal neurons. In each graph, the z-scored firing rates of SWRs are shown. **g** Comparison of the firing rates of ventral hippocampal SWRs between the prestress and poststress periods. Each dot represents a cell (*n* = 40 cells). **h** Relationship between the ratio of the firing rate in the SD period to that in the prestress period and the ratio of the SWR-associated firing rate in the poststress period to that in the prestress period. Each dot represents a neuron (*n* = 15, 5, and 20 neurons). **i** Distributions of the same data corresponding to H. An asterisk indicates a significant difference (*Z* = 2.78, *P = 0.0054, two-sided Mann–Whitney *U* test) in the SWR-associated firing rate between the prestress and poststress periods.

We next examined whether and how ventral hippocampal neurons encode SD experiences and ventral hippocampal SWRs reactivate these individual ventral hippocampal neurons in the poststress period (Fig. 3a, b). Figure 3c shows the changes in the spike rates of two putative pyramidal neurons in the CA1 region of the vHC during the SD period. Periods with massive noise signals due to strong physical contacts with an aggressor mouse (Fig. 3b) and fast running behavior (the yellow regions in Fig. 3c) were excluded from the analysis. The spike rate of neuron #1 was significantly increased during the SD period compared with the prestress period (*t*_29_ = 50.1, *P* < 10^−10^, Student’s *t test*); thus, neuron #1 was defined as an SD-excited neuron and was considered to encode SD episodes. In contrast, the firing rate of neuron #2 was significantly reduced during the SD period; thus, neuron #2 was termed an SD-inhibited neuron (*t*_29_ = –14.1, *P* < 10^−10^, Student’s *t test*). Examples of other ventral hippocampal neurons are shown in Supplementary Fig. 5. Of the 40 putative pyramidal neurons in the CA1 region of the vHC recorded, 37.5% and 50.0% of the neurons were classified as SD-excited neurons and SD-inhibited neurons, respectively (Fig. 3d and Supplementary Fig. 5a, b). The remaining fraction (12.5%) of neurons was classified as SD-insensitive neurons (Supplementary Fig. 5c). Overall, no significant differences in prestress (baseline) firing rates were detected among the SD-excited, SD-inhibited, and SD-insensitive pyramidal neurons (Supplementary Fig. 5d and 5e; *P* > 0.05, Mann–Whitney *U* test followed by Bonferroni correction). Of the 4 putative interneurons in the CA1 region of the vHC recorded, 3 and 1 neurons were classified as SD-inhibited neurons and SD-insensitive neurons, respectively (Supplementary Fig. 5d). The same types of cells were also observed from putative pyramidal neurons in the CA3 region of the vHC (Supplementary Fig. 5f).

Consistent with the poststress increase in the ventral hippocampal SWR rate (Fig. 2d, f), the firing rates of the majority of SD-excited cells continued to increase in the subsequent poststress period (e.g., Fig. 3c, left and Supplementary Fig. 5). Plotting of the average spike rates of all the neurons revealed that these increases were significant for 2 h after SD (Fig. 3e; *P* < 0.05, Mann–Whitney *U* test followed by Bonferroni correction). The firing rates of the SD-inhibited neurons showed no significant differences between the prestress and poststress periods (*P* > 0.05, Mann–Whitney *U* test followed by Bonferroni correction). These results demonstrate that neuronal ensemble patterns activated by an SD experience are preferentially reactivated in the subsequent rest period, indicating memory reactivation of the SD experience, and that this process may underlie poststress ventral hippocampal activity-dependent social behavior deficits.

To test how ventral hippocampal SWRs are associated with neuronal reactivation in the poststress period, ventral hippocampal SWR-triggered neuronal spikes were compared between the prestress and poststress periods (Fig. 3f, g). To evaluate the degree of poststress SWR-mediated reactivation, compared with that in the prestress period, we computed the ratio of the ventral hippocampal SWR-triggered-firing rate in the poststress period to that in the prestress period. Figure 3h summarizes all ratios from individual neurons plotted against the difference in the firing rate between the prestress and SD periods (i.e., the degree of encoding of SD experiences as a measure to define the SD-excited and SD-inhibited neurons). Overall, the SWR-associated firing rate of SD-excited neurons was significantly increased in the poststress period compared with the prestress period (Fig. 3i; *Z* = 2.78, *P* = 0.0054, Mann–Whitney *U* test), whereas there was no significant change in the firing rate of the SD-inhibited neurons (*Z* = 0.85, *P* = 0.39, Mann–Whitney *U* test). These results demonstrate that ventral hippocampal neurons that encode an SD experience are preferentially reactivated during ventral hippocampal SWRs in the subsequent poststress period.

### Ventral hippocampal SWRs affect amygdalar LFP activity

**Fig. 4:**
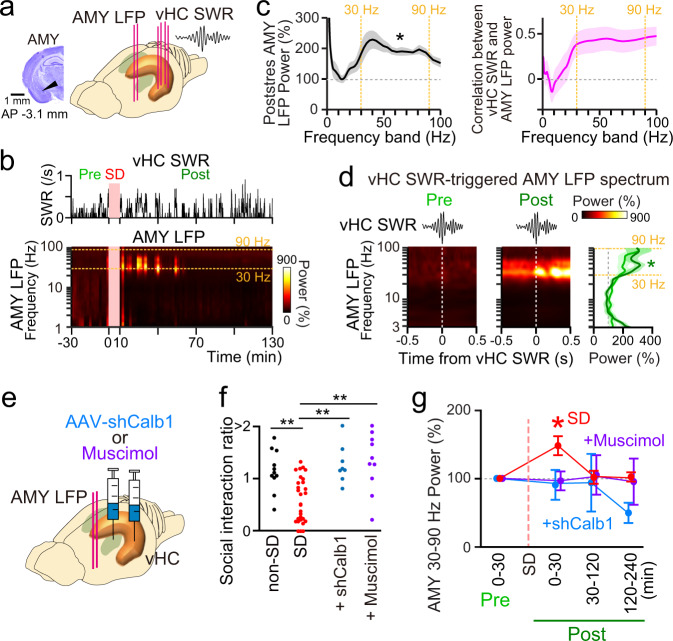
Interactions between ventral hippocampal SWRs and amygdalar LFP power related to SD stress. **a** Simultaneous recordings of LFP signals from the vHC and the AMY (histological confirmation shown in the left panel). The same results were obtained from 5 mice with electrodes implanted in the amygdala. **b** (Top) Changes in the ventral hippocampal SWR rate (bin = 2 min) in an SD mouse. The SD period was removed from the analysis. (Bottom) The corresponding amygdalar LFP power spectrum normalized to the average power during the prestress period in each frequency band. **c** (Left) Amygdalar LFP power in the 30-min poststress period normalized by the 0–30-min prestress, plotted against each frequency band (*n* = 5 mice). The asterisk indicates the 30–90 Hz band, in which there was a significant LFP power change between the two periods (*t*_4_ = 5.47, *P = 0.0055, two-sided paired *t test*). The data are the mean ± SEM. (Right) Correlations between the ventral hippocampal SWR rate changes and amygdalar LFP power changes in the poststress period. **d** (Left) Ventral hippocampal SWR-triggered AMY LFP power spectra in the prestress and poststress periods in an SD mouse. (Right) Average ventral hippocampal SWR-triggered amygdalar LFP power from all recordings (*n* = 5 mice) plotted against each frequency band. The asterisk indicates a significant increase in power in the 30–90 Hz band relative to 100%, as indicated by paired *t test* (*P* < 0.05). **e** LFP signals were recorded from the AMY in AAV-shCalb1-injected mice or mice that were bilaterally injected with muscimol into the vHC after the SD period. **f** SI ratios of non-SD mice (uninjected; *n* = 11 mice), SD mice (uninjected; *n* = 31 mice), AAV-shCalb1-injected mice (*n* = 8 mice, and SD mice injected with muscimol into the vHC (*n* = 10 mice). Non-SD versus SD, *Z* = 3.23, **P = 0.0036; SD versus shCalb1, *Z* = 3.15, **P = 0.0048; SD versus muscimol, *Z* = 3.26, **P = 0.0022, two-sided Mann–Whitney *U* test followed by Bonferroni correction. **g** SD-induced changes in the average AMY power in the 30–90 Hz band in uninjected SD mice (red, *n* = 5 mice), AAV-shCalb1-injected mice (cyan, *n* = 5 mice), and mice injected with muscimol into the vHC (purple, *n* = 4 mice). *P < 0.05, 0–30-min prestress versus 0–30-min poststress periods in the SD mice, two-sided Mann–Whitney *U* test followed by Bonferroni correction. The data are the mean ± SEM.

The vHC and amygdala reciprocally communicate with each other, working as an integrated system to regulate social and emotional memory^20,45,46^. While dorsal hippocampal SWRs have been shown to induce coordinated reactivation of hippocampal and amygdalar cells^47^, the contribution of ventral hippocampal SWRs, especially after emotional episodes, to amygdalar activity remains unknown. Thus, we obtained simultaneous LFP recordings from the vHC and basolateral amygdala (here, simply abbreviated as AMY) (Fig. 4a). Amygdalar LFP power spectra normalized to the average power in the prestress period are shown (bin = 2 min) (Fig. 4b). In the fast-gamma (60–100 Hz) bands, amygdalar LFP power in the poststress period was significantly higher than that in the prestress period, whereas such significance was not detected in the delta (1–4 Hz), theta (6–10 Hz), and slow-gamma (20–50 Hz) bands (Supplementary Fig. 6a; *n* = 5 mice, delta: *t*_4_ = 1.39, *P* = 0.24; theta: *t*_4_ = 0.56, *P* = 0.60; slow-gamma: *t*_4_ = 1.31, *P* = 0.26; fast-gamma: *t*_4_ = 2.84, *P* = 0.047, paired *t test*). Further inspections based on LFP power spectrograms ranging from 20–100 Hz (Supplementary Fig. 6b) confirmed that significantly higher amygdalar LFP power in the poststress period was observed specifically in the frequency bands of 30–90 Hz (Fig. 4c, left; *n* = 5 mice, *t*_4_ = 5.47, *P* = 0.0055, paired *t test*). No pronounced correlation was observed between increases in amygdalar 30–90 Hz power in the poststress period and subsequent SI ratios (Supplementary Fig. 6c; *r* = –0.11, *P* = 0.72).

The relationship of LFP signals between the vHC and AMY was analyzed by computing their coherence (Supplementary Fig. 6d). In all frequency bands, vHC-AMY LFP coherence was significantly higher than 0 (Supplementary Fig. 6e; *n* = 5 mice, paired *t* test, *q* < 0.05, FDR corrected) but no significant changes in coherence were observed between the prestress and poststress periods (Supplementary Fig. 6e; *n* = 5 mice, paired *t* test, *P* > 0.05). These results demonstrate that coherence of LFP signals between the vHC and AMY is not sensitive to SD stress. We next analyzed how ventral hippocampal SWRs are related to amygdalar LFP power and found that there was a significant positive correlation between the rate of ventral hippocampal SWRs and amygdalar 30–90 Hz LFP power (Fig. 4c, right; *n* = 5 mice, *t*_4_ = 3.38, *P* = 0.028, paired *t* test), suggesting a functional association between ventral hippocampal SWRs and amygdalar LFP patterns. To further examine the relationship at a higher temporal resolution, we constructed ventral hippocampal SWR-triggered amygdalar LFP spectra (Fig. 4d, left). The ventral hippocampal SWR-triggered amygdalar power in the 30–90 Hz range in the poststress period was significantly higher than the LFP power in the prestress period without SWRs (100–500 ms before and after SWRs) (Fig. 4d, right; *t*_4_ = 3.15, *P* = 0.035, paired *t* test), whereas such significance was not observed in the prestress period (*t*_4_ = 1.52, *P* = 0.20, paired *t* test). These results suggest that ventral hippocampal SWRs alter 30–90 Hz LFP patterns in the AMY and that this effect of ventral hippocampal SWRs becomes stronger after an SD experience. To further evaluate whether these amygdalar signals were associated with the Calb1 expression in the vHC, we recorded amygdalar LFP signals in AAV-shCalb1-injected mice (Fig. 4e) that showed significantly higher SI ratios, compared with non-injected SD mice, in a subsequent SI test on the next day (Fig. 4f; *n* = 8 mice, *Z* = 3.15, *P* = 0.0048, Mann–Whitney *U* test followed by Bonferroni correction). These AAV-shCalb1-injected mice showed no significant increase in amygdalar LFP power in the poststress period (Fig. 4g; *n* = 8 mice, *P* > 0.05, Mann–Whitney *U* test followed by Bonferroni correction). These results demonstrate the necessity of the Calb1 expression in SD-induced changes in amygdalar 30–90 Hz LFP power. Similar results were also observed from mice that were subjected to local injection of muscimol, a γ-aminobutyric acid (GABA)A receptor agonist, into the vHC immediately after SD stress exposure (Fig. 4f, behavior: *n* = 10 mice, *Z* = 3.26, *P* = 0.0022, Mann–Whitney *U* test followed by Bonferroni correction; Fig. 4g, amygdalar LFP power; *P* > 0.05, Mann–Whitney *U* test followed by Bonferroni correction), confirming the necessity of poststress ventral hippocampal activity in the induction of SD-induced increases in amygdalar 30–90 Hz LFP power and social behavioral deficits.

### Suppression of poststress ventral hippocampal SWRs rescues SI deficits

**Fig. 5:**
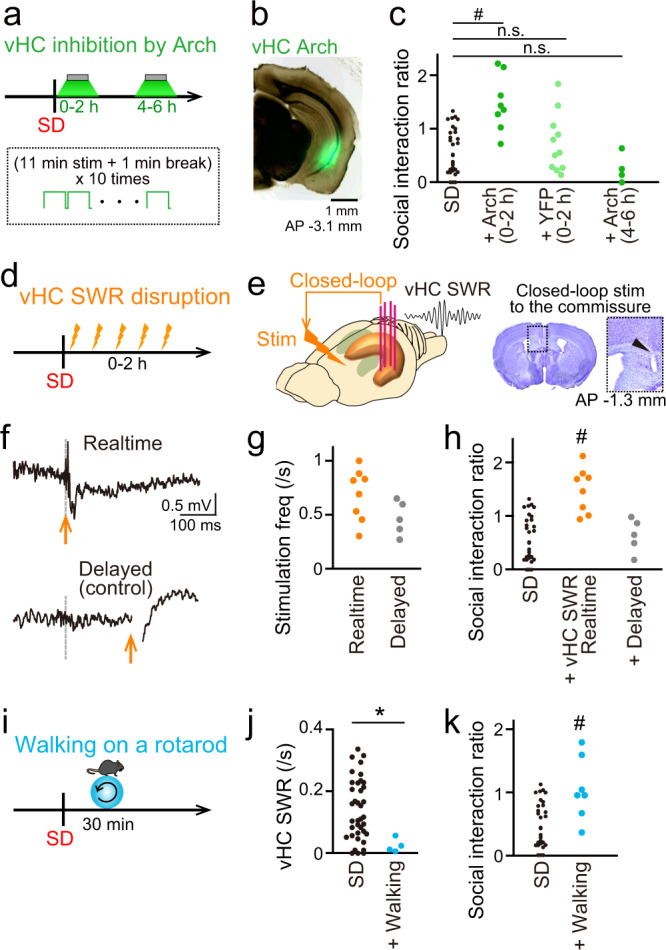
Suppression of poststress ventral hippocampal SWRs rescues SI deficits. **a** Experimental timeline of optogenetic inhibition (11-min green light laser pulses (λ = 532 nm; 1–2 mW) with intervals of 1 min) of the ventral hippocampus by Arch 0–2 h (Arch (0–2 h)) and 4–6 h (Arch (4–6 h)) after SD. **b** Arch-EYFP expression in the vHC. **c** SI ratios of SD mice after vHC Arch photostimulation (*n* = 31, 8, 11, and 4 mice). SD versus Arch(0–2 h), *Z* = 3.60, ^#^*P* = 6.3 × 10^−4^; SD versus YFP(0–2 h), *Z* = 0.66, *P* > 0.99; SD versus Arch(4–6 h), *Z* = 1.61, *P* = 0.22, two-sided Mann–Whitney *U* test followed by Bonferroni correction. The plots for the SD group are similar to those shown in Fig. 4g, which are presented for comparison. **d** Experimental timeline of closed-loop disruption of ventral hippocampal SWRs for 2 h after SD. **e** Histological verification of a stimulation electrode in the ventral hippocampal commissure. The dotted region is magnified in the right panel, and the electrode track indicated by the arrowhead. **f** A ventral hippocampal SWR detected at the gray dotted line was disrupted by real-time stimulation upon detection (top, arrow), whereas stimulation was delivered at 250 ms after detection in the control experiment (bottom, arrow). **g** The frequency of electrical stimulation applied in the real-time and delayed control groups (*n* = 8 and 5 mice). *P* = 0.093, two-sided Mann–Whitney *U* test. **h** SI ratios after ventral hippocampal SWR disruption (*n* = 31, 8, and 5 mice). The plots for the SD group are similar to those shown in c, which are presented for comparison. SD versus realtime, *Z* = 3.72, ^#^*P* = 3.9 × 10^−4^; SD versus delayed, *Z* = 0.09, *P* > 0.99, two-sided Mann–Whitney *U* test followed by Bonferroni correction. **i** Experimental timeline of forced walking for 30 min after SD. **j** The frequency of SWRs in the vHC during walking (*n* = 38 and 5 mice). *Z* = 2.59, *P = 0.0095, two-sided Mann–Whitney *U* test. **k** SI ratios after walking (*n* = 31 and 7 mice). The plots for the SD group are similar to those shown in c, which are presented for comparison. *Z* = 2.37, ^#^*P* = 0.012 versus SD mice, two-sided Mann–Whitney *U* test.

We further evaluated the causal role of poststress ventral hippocampal SWRs in SD-induced social interaction deficits. In following experiments, all experimental conditions and timelines (a 10-min SD stress and a SI test on the next day) were similar to those used for electrophysiological recordings, except that specific manipulations of neuronal activity or behavior were applied in the poststress periods. First, we confirmed the detailed time window of the ventral hippocampal activity-mediated effect by an optogenetic approach using archaerhodopsin-3.0 (Arch). Continuous 11-min green light laser pulses (*λ* = 532 nm; 1–2 mW) were repeatedly applied with intervals of 1 min for 2 h. Optogenetic inhibition of ventral hippocampal neurons for 0–2 h, but not 4–6 h, after SD exposure led to a significantly higher SI ratio (Fig. 5a–c; +Arch (0–2 h): *n* = 8 mice, *Z* = 3.60, *P* = 6.3 × 10^−4^; +Arch (4–6 h): *n* = 4 mice, *Z* = 1.61, *P* = 0.22, Mann–Whitney *U* test followed by Bonferroni correction). Such significant difference was not observed from mice expressing YFP alone (Fig. 5c, +YFP (0–2 h): *n* = 11 mice, *Z* = 0.66, *P* > 0.99, Mann–Whitney *U* test followed by Bonferroni correction). These results demonstrate that ventral hippocampal neuronal activity, especially for 2 h after stressful experiences, is crucial for inducing social behavior deficits. The time window corresponded with that of the significant increase in the vHC SWR rate (Fig. 2d). To selectively disrupt vHC SWRs in this time window, we implanted stimulation electrodes into the ventral hippocampal commissure (Fig. 5d, e) and delivered real-time feedback electrical stimulation with a single pulse (160–200 μA, 100 μs) upon the detection of vHC SWRs to transiently eliminate the vHC SWRs (Fig. 5f)^42,48^. The frequency of detected ventral hippocampal SWRs and real-time stimulation for 2 h in the poststress period was 0.68 ± 0.08 times per second (*n* = 8 mice, Fig. 5g). As a control experiment, the same stimulation was delivered 250 ms after the detection of vHC SWRs to leave the vHC SWRs intact. The frequency of delayed stimulation was 0.46 ± 0.10 times per second (*n* = 5 mice, Fig. 5g) and was not significantly different from that of real-time stimulation (*P* = 0.093, Mann–Whitney *U* test). Under these conditions, real-time disruption of ventral hippocampal SWRs during the 2-hour poststress period resulted in a significantly higher SI ratio (Fig. 5h; *n* = 8 mice, *Z* = 3.72, *P* = 3.9 × 10^−4^, Mann–Whitney *U* test followed by Bonferroni correction), whereas delayed stimulation did not induce such a significant effect (*n* = 5 mice, *Z* = 0.09, *P* > 0.99, Mann–Whitney *U* test followed by Bonferroni correction). These results demonstrate that the inhibition of ventral hippocampal SWR-related neuronal reactivation in the poststress period suppressed SD-induced social behavioral deficits.

In the poststress period, the SD mice were almost immobile in their home cages, which is an appropriate condition for generating ventral hippocampal SWRs that might exacerbate social behavior deficits. We next asked whether naturally reducing the rate of hippocampal SWRs can rescue social behavior deficits. As hippocampal SWRs are less frequent during active locomotion and exploration, during which theta (4–12 Hz) rhythms are more dominant, we assessed the effects of reducing the rate of ventral hippocampal SWRs by exercise on social behavior. Immediately after exposure to SD, seven mice were forced to walk for 30 min on a rotarod apparatus (3 rpm; Fig. 5i). All the mice successfully walked without falling from the rod. The ventral hippocampal SWR rate was significantly reduced to 0.019 ± 0.013 per second (*n* = 4 mice) in mice subjected to a 30-min walking period compared with SD mice (Fig. 5j; *Z* = 2.59, *P* = 0.0095, Mann–Whitney *U* test). The mice that walked after SD exhibited significantly higher SI ratios than the SD mice (Fig. 5k; *n* = 7 mice, *Z* = 2.37, *P* = 0.012, Mann–Whitney *U* test). In the hippocampus, theta states are dominant with fewer SWRs for memory encoding during movement (including walking), whereas SWRs preferentially occur for memory consolidation during immobility, rest, and sleep periods. Taken together with these behavior-relevant hippocampal memory processing modes, our results suggest that forced movement by walking after exposure to SD, during which memory consolidation in the vHC is almost absent, suppresses social behavior deficits.

## Discussion

The vHC receives hypothalamic and amygdalar afferents^45,49^, and ventral hippocampal neurons are specialized for encoding emotional and social components^15–17^, which can explain the importance of the vHC in stress susceptibility^5,16,28,29^. We addressed this issue at the levels of gene expression patterns and neuronal population dynamics in the vHC. Transcriptome analysis of ventral hippocampal tissue samples from stress-naïve mice demonstrated that Calb1 expression in the vHC is related to susceptibility to subsequent SD stress exposure. By manipulating Calb1 expression in the vHC, we found that SD stress specifically increased the rate of ventral hippocampal SWRs in stress susceptible mice but not in Calb1-deficient mice or stress-resilient mice. Poststress SWRs reactivated vHC pyramidal neurons that had encoded stress experiences and triggered 30–90 Hz power increases in the AMY. These results suggest that ventral hippocampal SWRs contribute to reactivation of memories of SD stress experiences in the ventral hippocampal-amygdalar circuit. Inhibition of ventral hippocampal SWRs by online feedback stimulation or by forced walking in the poststress period suppressed subsequent social behavior deficits. Taken together, our results suggest that Calb1-dependent memory reactivation processes in the vHC are a key mediator of stress susceptibility and the exacerbation of stress-induced neuropsychiatric symptoms (Supplementary Fig. 7).

Our analysis revealed that the gene expression of calcium signaling-related molecules such as Calb1 was higher in stress-naïve mice that exhibited higher susceptibility to SD stress exposure. As Calb1 is a calcium-binding protein that plays a central role in the regulation and buffering of intracellular calcium dynamics especially in dendrites and spines^50,51^, constitutive changes in Calb1 expression levels might affect calcium-dependent signaling cascades and neurotransmission, potentially leading to changes in overall activity patterns of hippocampal circuits. However, we found no significant changes in LFP power and the rate of SWRs by knockdown of Calb1 in the vHC under stress-naïve conditions, suggesting that neuronal population activity at basal states is maintained even when Calb1 expression is abolished. Further examinations are required to determine whether this maintenance under Calb1 knockdown is results of compensation mechanisms such as the regulation of other functional molecules and adaptive plasticity of neurotransmission and whether changes in the other neurophysiological dynamics at the cellular and synaptic levels occur at basal states. On the other hand, we demonstrated that the knockdown of Calb1 expression affected stress-induced increases in SWRs in the vHC, suggesting that Calb1 expression is necessary to create neuronal population activity induced by novel stressful episodes. This change in network-level neuronal activity is likely triggered by Calb1-dependent synaptic plasticity and neuronal excitability^38,52^ that should inherently occur after stressful episodes. Moreover, the knockdown of Calb1 expression led to decreases in ventral hippocampal SWRs, leading to subsequent social interaction deficits. This result is consistent with behavioral studies that Calb1 knockdown in the hippocampus disrupts spatial memory^38,53^, demonstrating the contribution of Calb1 in memory mechanisms. Taken together, ventral hippocampal Calb1 is considered as a crucial factor for animal’s adaptive behavior in response to novel aversive episodes such as stressful experiences by supporting the formation of memory-related neuronal ensembles. Originally, such Calb1-dependent hippocampal memory processes may have been advantageous for animal survival by increasing the probability of avoiding danger. On the other hand, our results provide an implication that they have the potential to exacerbate stress-induced psychiatric symptoms if they occur excessively after negative stressful experiences.

A well-established theory of memory suggests that learned information needs to be consolidated into neuronal circuits to establish long-term memory by repeated reactivation of memory-encoding neuronal activity^54,55^. Hippocampal SWRs play a central role in memory consolidation during the postexperience period^40–42,56^. Our results suggest that stress experiences are more strongly consolidated in the ventral hippocampal circuit, almost independently from the dorsal hippocampal circuit. The strong excitation gain of ventral hippocampal neurons by SWRs is considered effective for transmitting information to downstream brain areas such as the NAc, mPFC, and AMY^15,17,28,44^. Especially, our results showed that ventral hippocampal SWRs more strongly impacted LFP power in the AMY in the poststress period than in the prestress period, suggesting that SD experiences strengthen vHC-AMY functional connections upon vHC SWRs. The enhancement of functional hippocampus-AMY connectivity is consistent with a study of depressed patients^57^. In addition, we showed that inhibition of ventral hippocampal activity abolished the SD-induced increase in amygdalar LFP power. These results suggest that information transfer through the ventral hippocampal-amygdalar circuit and memory consolidation within the ventral hippocampal circuit occur concurrently and that both are primarily promoted by ventral hippocampal SWRs. Considering the distinct roles of the hippocampus in processing contextual memory and the AMY in the regulation of fear and anxiety-related behavior, it is conceivable that ventral hippocampal SWRs link these two functions to cooperatively determine the degree of stress memory-induced psychiatric symptoms. Finally, we showed that dynamic perturbation of these ventral hippocampal SWRs by online stimulation, possibly inhibiting memory consolidation of SD episodes, was effective in suppressing SD-induced social behavior deficits. In addition to the vHC-AMY connections, vHC-NAc connections have been shown to be crucial to determine stress-induced social behavioral deficits. Activation of the vHC-NAc pathway induced a stress-susceptible effect, whereas depressing this pathway induced a stress-resilient effect^28^. Furthermore, individual differences in baseline activity of vHC neurons projecting to the NAc predict intrinsic susceptibility to stress experiences^58^. As our study did not test the involvement of the vHC-NAc projections, further studies are necessary to test whether the stress-induced memory reactivated by ventral hippocampal SWRs is further read out and processed by the NAc.

The roles of hippocampal SWRs in memory processing have been mainly studied from the perspective of their positive aspects, such as strengthening of learned memory for achieving efficient future behavior^42,47^. On the other hand, this study highlighted a negative aspect of a hippocampal SWR-associated memory processes: increased reactivation of stressful memory that potentially amplifies stress susceptibility. These results are consistent with the idea that not only external stressful events but also internal representation (recall) of negative emotional and stressful memory are crucial for the development of mental changes^12,57,59,60^. Given that hippocampal SWRs serve as a common substrate for memory retrieval^56^ and simulated experiences in both animals and humans^48,61^, stress-related hippocampal SWRs might be involved in human psychopathology such as depressive rumination—the tendency of repetitive recall of negative memory and affect^12,57,59,60^. As shown in our study, interrupting vHC memory reactivation, such as through exercise, may be an effective therapeutic intervention for stress-induced mood disorders.

## Methods

### Approvals

All experiments were performed with the approval of the animal experimental ethics committee at the University of Tokyo (approval number: P29-14) and the committee on animal experiments at Tohoku University (approval number: 2022 PhA-004) and in accordance with the NIH guidelines for the care and use of animals.

### Subjects

C57BL/6 J mice (10–15 weeks old) with preoperative weights of 22–35 g were used as intruder mice that received social defeat (SD) stress or as control mice. Mice were housed on a 12-h light/12-h dark schedule with lights off at 8:00 PM. In addition, CD-1 mice (more than 13 weeks old) with weights of 40–50 g were used as resident mice that imposed social defeat stress. They were individually housed and maintained on a 12-h light/12-h dark schedule under housing conditions at 23 ± 1 °C with relative humidity of 50 ± 5% with lights off at 8:00 AM. All mice were purchased from SLC (Shizuoka, Japan).

### Generation of AAV shRNA

Recombinant adeno-associated viruses (AAVs) were generated by triple transfection of the 293 AAV cell line (AAV-100; Cell Biolabs, Inc., San Diego, CA) with AAVdj rep-cap, pHelper from the AAV-DJ Helper Free Packaging System (VPK-400-DJ; Cell Biolabs, Inc.) and pAAV-U6-shRNA-CMV-mCherry using PEI-Max (24765; Polysciences, Inc., Warrington, PA). AAV vectors were purified using the AAVpro Purification Kit All Serotypes (6666; Takara Bio Inc., Shiga, Japan). Virus titers were determined by qPCR using the AAV2 ITR primer pair^62^, THUNDERBIRD Next SYBR qPCR Mix (QPX-201; TOYOBO, Osaka, Japan), and the LightCycler qPCR 2.0 system (DX400; Roche, Basel, Switzerland). The AAV shRNA hairpin sequences were Calb1-shRNA ^53^: 5′-GCT GGA TGC TTT GCT GAA AGA CTC GAG TCT TTC AGC AAA GCA TCC AGC TTT TT-3′ and Scramble-shRNA^63^: 5′-GCA TAC GGT CAA TCC TCA ACA CTC GAG TGT TGA GGA TTG ACC GTA TGC TTT TT-3′.

### Surgical procedures

In all surgeries, mice were first anesthetized with isoflurane gas (1–3%) and a midline incision was made from the area between the eyes to the cerebellum. Craniotomies with a diameter of 1.0–1.4 mm were created above target brain regions using a high-speed drill, and the dura was surgically removed.

For tissue collection, a unilateral stainless guide tube (3.4 mm length, 1.1 mm inner diameter (I.D.), 1.5 mm outer diameter (O.D.)) was implanted into the right ventral hippocampus (3.1 mm posterior and 3.6 mm lateral to bregma, depth of 3.5 mm). A wire with the same length as the guide tube and with the tip having a cross shape (1.1 mm diameter) was then inserted into the guide tube. By rotating the wire and pulling it out, the small brain area attaching to the tip was removed. This procedure cleaned the area above the tissue to be collected. Next, a wire with the tip having a cross shape (1.1 mm diameter) that protruded from the guide tube by 1 mm was inserted into the guide tube. By rotating the wire and pulling it out, a tissue sample of the ventral hippocampus was collected.

For virus injection, glass pipettes (ϕ = 30–40 µm) were bilaterally inserted into both sides of the ventral hippocampus (3.1 mm posterior and ±3.6 mm lateral to bregma, depth of 3.5 mm) and AAVdj-shCalb1-mCherry (1.14 × 10^14^ vg/ml)^53^, AAVdj-shScramble-mCherry (1.07 × 10^13^ vg/ml)^63^, and AAVdj-Calb1^OE^-GFP (3.4 × 10^12^ vg/ml) dissolved in phosphate-buffered saline (PBS; pH 7.4) was injected at a rate of 100 nl/min for 3 min. After the injection, the injection pipette was left in place for 1 min and then raised 50 µm and again left in place for 5 min.

For drug injection, guide tubes (inner diameter = 0.34 mm and outer diameter = 0.5 mm) were bilaterally implanted into both sides of the dorsal (1.8 mm posterior and ±1.8 mm lateral to bregma, depth of 1.5 mm) or ventral (3.1 mm posterior and ±3.6 mm lateral to bregma, depth of 3.5 mm) hippocampus. To prevent drying in the guide tubes, a dummy plastic cannula with a diameter of 0.33 mm was inserted into the guide tube until the muscimol injection described below.

For optogenetic experiments, glass pipettes (ϕ = 30–40 µm) were bilaterally inserted into both sides of the ventral hippocampus (3.1 mm posterior and ±3.6 mm lateral to bregma, depth of 3.5 mm) and AAV5-CaMKII-Arch3.0-EYFP or AAV5-CaMKII-EYFP (the UNC Vector Core service) dissolved in phosphate-buffered saline (PBS; pH 7.4) was injected at a rate of 100 nl/min for 3 min. After the injection, the injection pipette was left in place for 1 min and then raised 50 µm and again left in place for 5 min. After removing the pipette, optical fibers (ϕ = 200 µm) were implanted at the same coordinates.

For LFP recordings without spikes, a tetrode assembly created using a 3D printer (Form 2, Formlabs)^64–66^ was directly implanted into (i) the right ventral hippocampus (3.1 mm posterior and 3.6 mm lateral to bregma, depth of 3.5 mm, 3 tetrodes), (ii) both the right dorsal hippocampus (1.5 mm posterior and 2.0 mm lateral to bregma, depth of 1.5 mm, 3 tetrodes) and ventral hippocampus (3.1 mm posterior and 3.6 mm lateral to bregma, depth of 3.5 mm, 3 tetrodes), and (iii) the ventral hippocampus (4 tetrodes) and the amygdala (0.8 mm posterior and 3.0 mm lateral to bregma, depth of 4.4 mm, 3 tetrodes). For all recordings, an electrode was additionally implanted into the somatosensory cortex (i and ii; 3.1 mm posterior and 3.6 mm lateral to bregma, a tetrode at a depth of 0.5 mm) or prefrontal cortex (iii; 2.0 mm anterior and 0.8 mm lateral to bregma, a stainless-steel screw electrode on the brain surface) to serve as reference electrodes. Two stainless-steel screws were implanted in the bone above the cerebellum to serve as ground electrodes. The tetrodes were constructed from 17-μm-wide polyimide-coated platinum-iridium (90/10%) wires and the tetrodes tips were plated with platinum to lower electrode impedances to 150–300 kΩ at 1 kHz.

For spike recordings, an electrode assembly that consisted of 6 independently movable tetrodes was stereotaxically implanted above the ventral hippocampus (3.1 mm posterior and 3.6 mm lateral to bregma) at a depth of 2.0 mm. The tetrodes were advanced to the targeted brain regions over a period of at least one week following surgery (for more detail, see Adjusting electrode depth as described below). For some mice, an additional incision was made at the incised neck area, and one EMG electrode was sutured to the dorsal neck muscles.

For closed-loop feedback stimulation, in addition to implantation of a tetrode assembly into the ventral hippocampus, stainless bipolar electrodes with an impedance of 3 MΩ were implanted into the right side of the ventral hippocampal commissure (0.7 mm posterior and 0.5 mm lateral to bregma, depth of 1.7 mm).

All device was secured to the skull using stainless-steel screws and dental cement. After all surgical procedures were completed, anesthesia was discontinued, and the mice were allowed to awaken spontaneously. Following surgery, each animal was housed in a transparent Plexiglas cage with free access to water and food for at least one week.

### Social defeat stress

C57BL/6J mice were exposed to SD stress^1,37^. At least 1 week before beginning the social defeat experiment, all resident CD-1 mice more than 13 weeks of age were singly housed in a home cage. The bedding in the resident area was left unchanged during the period. First, resident CD-1 mice were screened as aggressors for social defeat experiments by introducing an intruder C57/BL6J mouse that was specifically used for screening into the home cage during three 3-min sessions on 2 subsequent days. Each session included a different intruder mouse. CD-1 mice were selected as aggressors in subsequent experiments based on three criteria: during the three 3-min sessions, (i) the mouse attacked in all three sessions, (ii) the latency to initial aggression was less than 20 s, and (iii) the above two criteria were met for the two consecutive days. This screening was especially crucial to select strong aggressor mice that could trigger behavioral deficit with a single stress load. After screening, an intruder C57BL/6J mouse was exposed to SD stress by introducing it into the home cage of the resident mouse with a light intensity of 20 lux for a 7–10-min interaction, termed a SD mouse. The interaction period was immediately terminated if the SD mouse had a wound and bleeding due to the attack, resulting in interaction periods of 7–10-min. After the physical contact, the SD mouse was transferred to a half compartment of a home cage of a resident mouse (42.5 cm × 26.6 cm × 15.5 cm) that was evenly divided by a Plexiglas partition (0.5 cm × 41.8 cm × 16.5 cm) with perforated holes, each with a diameter of 10 mm. The second resident mouse was placed in the opposite compartment for the following 24 h; this allowed the SD mouse to have sensory contact with the resident mouse without physical contact. Over the following 10-day period, the intruder mouse was exposed to a new resident mouse so that the animals did not habituate the same residents, unless otherwise specified. This general protocol of SD stress was applied to mice that underwent virus injection as in Fig. 1h and Supplementary Fig. 1b.

In Fig. 1a–f, mice that underwent vHC tissue sample collection were subjected to SD stress every 2 days for 10 days to reduce postsurgery physical damage. Similar to daily SD stress (e.g. Fig. 1h and Supplementary Fig. 1b), this protocol was sufficient to yield susceptible and resilient mouse types.

In Figs. 2–5, mice received a surgery and were implanted with recording device, injection cannula, or optical fibers on the brain. These mice were subjected to SD stress for 1 day and placed back to their own home cage and singly housed without aggressors. The next day, the mice were tested in a SI test (as described later). To more strongly induce the effects of 1-day stress responses, all SI tests were performed in the same room and same location and with the same experimental condition so that SD mice more strongly recalled an episode of SD stress.

When electrophysiological recordings were performed from the SD mouse, the mouse was first transferred to its home cage and electrophysiological signals were obtained for several hours after SD stress. After recordings, the SD mouse was transferred in the opposite compartment of the second resident home cage for the following 20–24 h, as described above.

In a non-SD group, C57BL/6J mice were housed for 10 min in the same home cage but subjected to no physical contact with a CD-1 mouse by partitioning the cage with a transparent wall (termed non-SD mice).

As a control experiment, mice were placed in a novel open field (39.3 cm × 39.3 cm) without any aggressor mice for 10 min (Supplementary Fig. 2d–f).

Immediately after receiving SD stress, some mice were placed on a rotarod apparatus with a constant rotation speed of 3 rpm and forced to walk for 30 min (walking group).

### Social interaction test

Social interaction (SI) tests were performed in a square-shaped box (39.3 cm × 39.3 cm) enclosed by walls 27 cm in height with a light intensity of 20 lux. A PLA-mesh cage (6.5 cm × 10 cm × 24 cm) was centered against one wall of the arena during all social interaction sessions. Each SI test included two 150-s sessions (separated by an intersession interval of ~30 s) without and with the target CD-1 mouse present in the mesh cage; these sessions were termed no target and target sessions, respectively. In the no target session, a C57BL/6J mouse was placed in the box and allowed to freely explore the environment. The C57BL/6J mouse was then removed from the box. During the ~30-s break between sessions, the target CD-1 mouse was introduced into the mesh cage. The design of the cage allowed the animal to fit its snout and paws through the mesh cage but not to escape from the cage. In the target session, the same C57BL/6J mouse was placed beside the wall opposite to the mesh cage. In each session, the time spent in the interaction zone, a 14.5 cm × 26 cm rectangular area extending 8 cm around the mesh cage was analyzed. The social interaction (SI) ratio, or simply termed as “social interaction” in figures, was computed as the ratio of time spent in the interaction zone in the presence of the target (target session) to the time spent there in the absence of the target (no target session).

### Local infusion of muscimol

Drug injection was performed in a home cage. The dummy cannula was removed from the guide tube and replaced by a plastic injection cannula with a diameter of 0.34 mm so that the tip of the injection cannula reached above the hippocampus. The other side of the injection cannula was connected by polyethylene tubing to a 50-μl syringe mounted in an infusion pump (KDS LEGATO101, Muromachi, Japan). Through the injection cannula, 1.0 μg/μl muscimol dissolved in phosphate-buffered saline (PBS; pH 7.4) was then infused for 5 min into the hippocampus at a rate of 100 nl/min. After the infusions were completed, the injection cannula was left in place for 5 min. Then, the dummy cannula was again inserted into the guide tube. During the muscimol injection procedure, the animals did not show any sign of stress or discomfort. As a control experiment, saline was injected with the same procedure.

### Photostimulation

Photostimulation was delivered to both sides of the ventral hippocampus. Continuous 11-min green light laser pulses (*λ* = 532 nm; 1–2 mW; COME2-LB473/532, Lucir, Japan) with intervals of 1 min were repeatedly applied ten times (in total, 2 h). The intervals were set to avoid phototoxicity due to long continuous photostimulation.

### Adjusting electrode depth

The mouse was connected to the recording equipment via Cereplex M (Blackrock), a digitally programmable amplifier, close to the mouse’s head. The output of the headstage was conducted via a lightweight multiwire tether and a commutator to the Cerebus recording system (Blackrock), a data acquisition system. Electrode turning was performed while the mouse was resting in a pot placed on a pedestal. The electrode tips were advanced slowly 25–200 μm per day for 10–20 days until spiking cells were encountered in the cell layer of the ventral hippocampus, which was identified on the basis of local field potential (LFP) signals and single-unit spike patterns. Once the tetrodes were adjacent to the cell layer, as indicated by the presence of multiunit activity, tetrodes were settled into the cell layer for stable recordings and recording commenced as described below.

### Electrophysiological recording

For recording electrophysiological signals, the EIB of the microdrive array was connected to a Cereplex M digital headstage (Blackrock Microsystems), and the digitized signals were transferred to a Cereplex Direct data acquisition system (Blackrock Microsystems). Electrical signals were sampled at 2 kHz and low-pass filtered at 500 Hz. The unit activity was amplified and bandpass filtered at 750 Hz to 6 kHz. Spike waveforms above a trigger threshold (60 μV) were time-stamped and recorded at 30 kHz in a time window of 1.6 ms.

Recordings were continuously performed for up to 1 h before SD stress, 10 min during SD stress, and for up to 4 h after SD stress. In 3 of the 10 mice, a recording cable was disconnected during the 10-min SD session due to severe attack from the resident mouse. In that case, immediately after applying SD stress, the intruder defeated mouse was located inside a mesh-caged cylinder (ϕ = 10 cm, height = 20 cm) located at the center of an open field (39.3 cm × 39.3 cm × 27 cm). The resident mouse was then placed outside the cylinder and allowed to freely explore within the open field with interacting the intruder mouse through the cylinder for 5 min, termed a forced interaction period, which allowed us to stably record neuronal activity that encoded SD episodes without noise due to physical attacks. These spike signals were also considered to encode SD experiences and integrated into our analysis.

### Video monitoring

In the SD period, all behavioral patterns of mice were monitored at 15 Hz using a video camera attached to the ceiling. In the SI test, the animal’s moment-to-moment position was recorded at 15 Hz using the CMOS camera (MCM4350, Gazo) attached on the ceiling. The frame rate of the movie was downsampled to 3 Hz, and the instantaneous speed of each frame was calculated based on the distance traveled within a frame (~333 ms). Animal’s trajectories in images were manually extracted by Image J1.45.

### Closed-loop electrical stimulation

Closed-loop electrical stimulation upon online detection of vHC SWRs was performed using extension codes implemented on the Cerebus recording system (Blackrock) and custom-made C + + codes^48^. A tetrode implanted into the vHC was chosen and the envelop of its band-pass (100–400 Hz) filtered LFP signals was computed in real time. vHC SWRs were detected online when the envelop exceeded the detection threshold of 3 standard deviations above the mean computed from LFP signals during stop periods in the rest box. At the time of vHC SWR detection, an electrical pulse with a duration of 100 μs and an amplitude of 160–200 μA was applied to the vHC. Stimulation rate was limited to a maximum of 4 Hz. For delayed control stimulation, stimulation was applied with a latency of 250 ms after the onset of ripple detection so that the stimulation occurred outside the detected vHC SWRs.

### Cresyl violet staining and immunohistochemistry

The mice were overdosed with urethane, perfused intracardially with 4% paraformaldehyde in PBS and decapitated. After dissection, the brains were fixed overnight in 4% PFA and equilibrated with 20% and 30% sucrose in phosphate-buffered saline for an overnight each. Frozen coronal sections (50 μm) were cut using a microtome, and serial sections were mounted and processed for cresyl violet staining. For cresyl violet staining, the slices were rinsed in water, counterstained with cresyl violet, and coverslipped with hydrophobic mounting medium (Marinol). The positions of all tetrodes were confirmed by identifying the corresponding tetrode tracks in histological tissue by using an optical microscope (All-in-One Fluorescence Microscope BZ-X710, Keyence Corporation, Osaka, Japan). In some animals, the tetrode tips were further advanced after recordings by passing the cell layer to confirm no cells were observed. In these cases, we estimated the location of tetrode tips at recording time based on records of the advancement of tetrodes during turning periods. For immunohistochemistry, the slices were rinsed with PBS and then permeabilized in 100 mM PBS with 0.3% Triton X-100 and 5% bovine serum albumin (BSA, 01863-77; Nacalai Tesque, Kyoto, Japan) at room temperature for 60 min. The slices were then incubated with a primary rabbit anti-NeuN antibody (1:2000, ab177487, Abcam, UK), primary goat anti-Calbindin antibody (1:400; Calbindin-Go-Af1040, Frontier Institute Co., Ltd., Hokkaido, Japan) in 100 mM PBS with 0.3% Triton X-100 and 5% BSA for one overnight period at 4 °C. After rinsing with PBS, they were then labeled with a secondary anti-rabbit IgG antibody Alexa 647 (1:1000; Thermo Fisher Scientific, Tokyo, Japan), anti-Goat IgG antibody Alexa 488 (1:1000; Thermo Fisher Scientific, Tokyo, Japan) in 100 mM PBS with 0.3% Triton X-100 and 5% BSA for 90 min. Images were acquired at a Z-depth interval of 0.775 μm using a confocal laser-scanning microscope (A1-HD25; Nikon, Tokyo, Japan) with an objective lens (×10, 0.45 NA; ×20, 0.75 NA).

### Microarray experiments and data analysis

The collected tissue sample was immediately frozen in liquid nitrogen. Total RNA was prepared from each of the mice using a RNeasy Plus Mini Kit (Qiagen Inc., Valencia, CA, USA). The samples with a concentration of 100 pg/μl were used for analysis. RNAs were applied to microarray analysis performed by Affymetrix GeneChip Mouse Clariom S arrays (Kurabo Industries Ltd., Osaka, Japan). The prepared microarrays were preprocessed with Transcriptome Viewer (Kurabo Industries Ltd., Osaka, Japan). Raw signals were transformed to the log2 scale and then normalized. In cases where the probes for a given gene yielded a p-value (detection p-value) greater than 0.05, the gene was excluded from further analysis. Differentially expressed genes (DEGs) were identified with a false discovery rate-adjusted p-value cutoff of 0.05 (one-sided Student’s *t*-test). The expressions of significant DEGs were compared between susceptible and resilient mouse types. The functions of DEGs were classified by gene ontology (GO) analysis.

### Real-time quantitative reverse transcription‑polymerase chain reaction

cDNA synthesis by ReverTra Ace (TOYOBO Co., Ltd.) was followed by qPCR with PowerUp SYBR Green Master Mix (Thermo Fisher Scientific) performed in 8-strip tubes on the Roche LightCycler® 96 thermocycler. The reaction conditions: 95 °C for 120 s, followed by 40 cycles (95 °C for 15 s, 60 °C for 60 s). Commercially available primers for the target gene, Calb1 (forward: 5′-CTTGCTGCTCTTTCGATGCCAG-3′, reverse: 5′-GTTCCTCGGTTTCGATGAAGCC-3′) and internal control gene, *Actb* (forward: 5′- GGCTGTATTCCCCTCCATCG −3′, reverse: 5′- CCAGTTGGTAACAATGCCATGT −3′) was obtained from Eurofins. The results were calculated by 2^−ΔΔCt^ method. Calb1 expression levels were normalized by those from stress resilient mice in each experiment.

### Spike sorting

Spike sorting was performed offline using the graphical cluster-cutting software MClust4.3.02^67^. Rest recordings before and after the behavioral paradigms were included in the analysis to assure recording stability throughout the experiment and to identify hippocampal cells that were silent during behavior. Clustering was performed manually in 2D projections of the multidimensional parameter space (i.e., comparisons between waveform amplitudes, the peak-to-trough amplitude differences, waveform energies, and the principal components of waveforms, each measured on the four channels of each tetrode). Cluster quality was measured by computing the *L*_ratio_ and the isolation distance^68^. The *L*_ratio_ was computed by the original equation, proposed by Schmitzer-Torbert et al. (2005), not normalized by the total number of spikes recorded on the tetrode. A cluster was considered as a cell when the *L*_ratio_ was less than 0.20 and the isolation distance was more than 15 (average *L*_ratio_ was 0.122 ± 0.009 and average isolation distance was 21.4 ± 0.8 in 36 isolated cells). In the auto-correlation histograms, cells with no clear refractory period (<3 ms) were excluded from analyses. In addition, in the cross-correlation histograms, putative cell pairs with a symmetrical gap around the center bins were considered to arise from the same cell and were merged. Finally, cells with spike waveforms longer than 300 μs and an average firing rate of <3 Hz throughout an entire recording period were considered putative pyramidal cells. On the other hand, cells with an average firing rate of more than 3 Hz throughout an entire recording period were considered putative interneurons.

### Detection of SWRs

A hippocampal LFP signal was bandpass filtered at 150–250 Hz, and the root mean-square power was calculated in the ripple-band with a bin size of 10 ms. SWR events were detected if the power exceeded a threshold for at least 15 ms. The threshold for SWR detection was set to 3 standard deviations (SDs) above the mean of all envelopes computed from the prestress periods. When an EMG signal from the dorsal neck muscle was simultaneously recorded, the signal was bandpass filtered at 20–200 Hz, and its root mean-square power was calculated with a bin size of 1 s. Periods with EMG power exceeding (2 × *Power*_mode_−*Power*_low_) were regarded as massive movement and excluded from this detection analysis, where *Power*_mode_ and *Power*_low_ represent the power giving the peak in the frequency distribution and the lowest power, respectively. When an EMG signal was not recorded, the same analysis for excluding periods with massive movement was applied to a LFP signal from a reference electrode placed in the neocortex.

### Spike rate analysis

To remove periods with massive electrical noise due to severe physical attack during a SD period, we computed the root mean-square power of prefrontal LFP signals with a bin size of 1 s as a reference signal including physical noise. Periods with transient huge increases in the power were manually detected from the power trace and average spike rate during SD periods were computed without these periods in each cell. In the prestress and poststress periods, instantaneous spike rates were computed with a bin size of 1 min. In each neuron, a neuron was considered significantly modulated (SD-excited or SD-inhibited) by SD stress if its average firing rate in a SD period was significantly higher or lower than a series of firing rates (bin = 1 min) in a prestress (baseline) period, respectively, defined by a paired *t*-test.

In the prestress and poststress periods, vHC SWR-triggered-firing rate changes were computed from all times of vHC SWRs observed in each rest period with a bin size of 20 ms. To compare the degree of SWR-related spike rate changes between the prestress and poststress periods, in each neuron, the triggered-firing rates were converted to z-scored firing rates based on the mean and SD of spike rate changes 100–500 ms before the vHC SWRs in the prestress period.

### LFP power analysis

LFP power at frequency bands of 1–100 Hz was computed by a Fourier transformation analysis. To compute the time-frequency representation of LFP power, amygdalar LFP signals were subtracted from prefrontal LFP signals and downsampled to a sampling rate of 200 Hz. The downsampled LFP traces were convolved using complex Morlet wavelet transformation at frequencies ranging from 1 to 100 Hz using a Matlab function. The absolute power spectrum of the LFP during each 10-ms time window was calculated and the power at each frequency band was normalized by the corresponding power averaged over the 0–30-min prestress period. Coherence between two electrodes was computed using a Wavelet coherence by the Matlab with a sampling rate of 200 Hz.

### Statistical analysis

All electrophysiological data are presented as the mean ± standard error of the mean (SEM) and were analyzed using MATLAB2019b. For behavioral data, comparisons of two-sample data were analyzed by Mann–Whitney *U* test or Wilcoxon signed rank test. Multiple group comparisons were performed by post hoc Bonferroni corrections. For Calb1 expression data, one sample with an extreme value was excluded based on Grubbs test. For spike-rate and SWR-rate data, comparisons of two-sample data were analyzed by paired *t*-test. Multiple group comparisons were performed by post hoc Bonferroni corrections. The null hypothesis was rejected at the *P* < 0.05 level.

### Reporting summary

Further information on research design is available in the Nature Portfolio Reporting Summary linked to this article.

## Supplementary information

Supplementary Information

Reporting Summary

## Data Availability

The original gene expression data are deposited in DDBJ Genomic Expression Archive (GEA) under accession number E-GEAD-490. Original physitological datasets are provided on Mendeley Data (https://data.mendeley.com/datasets/7jdtbdx2gp/1). Source data are provided with this paper.

## Source data

Source Data

## Code availability

Original codes are provided on Mendeley Data (https://data.mendeley.com/datasets/7jdtbdx2gp/1). MClust software is available from A.D. Redish at https://redishlab.umn.edu/mclust.
