# Supplementary material for: Hippocampal sharp wave ripples underlie stress susceptibility in male mice
Source: Nat Commun. 2023 Apr 20;14:2105. doi: 10.1038/s41467-023-37736-x (PMC10119298; doi:10.1038/s41467-023-37736-x)
Supplement: Supplementary file 1 — Supplementary Information [file 41467_2023_37736_MOESM1_ESM.pdf]

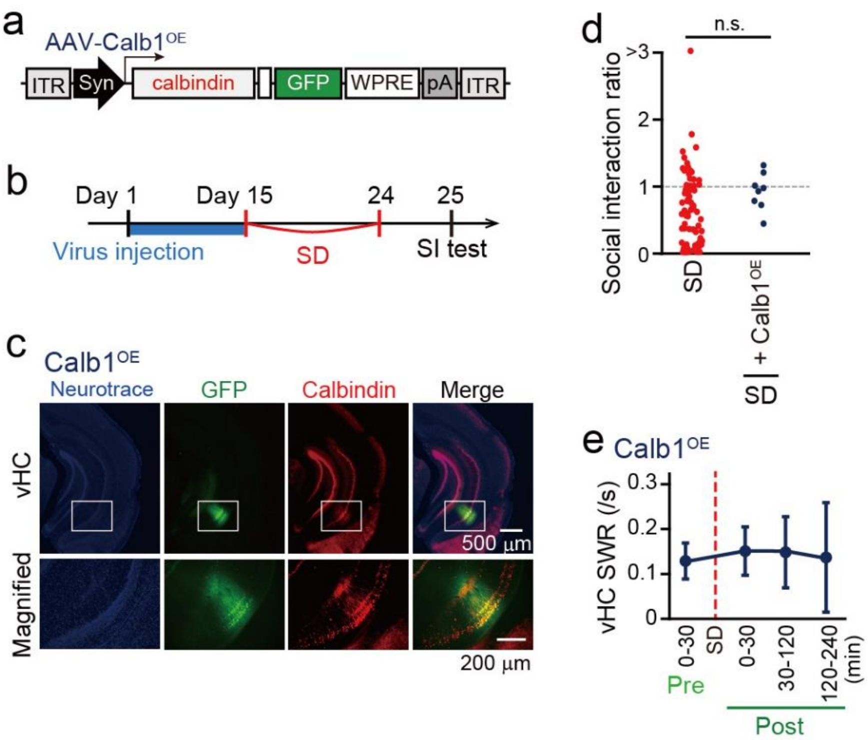

Fig. S1: The effect of overexpression of Calb1 on poststress social interaction and ventral hippocampal SWRs.

a Structures of the AAV construct for Calb1 overexpression. **b** Experimental timeline of virus injection, similar to Figure 1h. **c** Representative images showing the overexpression of Calb1 (red) and GFP (green) in the vHC in AAV-Ca1b1^{OE}-injected mice. The while boxes in the top panels are magnified in the bottom panels and show overexpression of Calb1 in GFP-expressing ventral hippocampal neurons. **d** SI ratios of SD mice and SD mice injected with AAV-Ca1b1^{OE} into the vHC (n = 73 and 8 mice). Each dot represents one mouse. The plots for the SD group are similar to those shown in Figure 1k, which are presented for comparison. $P > 0.05$ versus SD mice, two-sided Mann–Whitney U test followed by Bonferroni correction. **e** Changes in the rates of ventral hippocampal SWRs in AAV-Ca1b1^{OE}-injected mice (black, n = 3 mice). The data are the mean ± SEM.

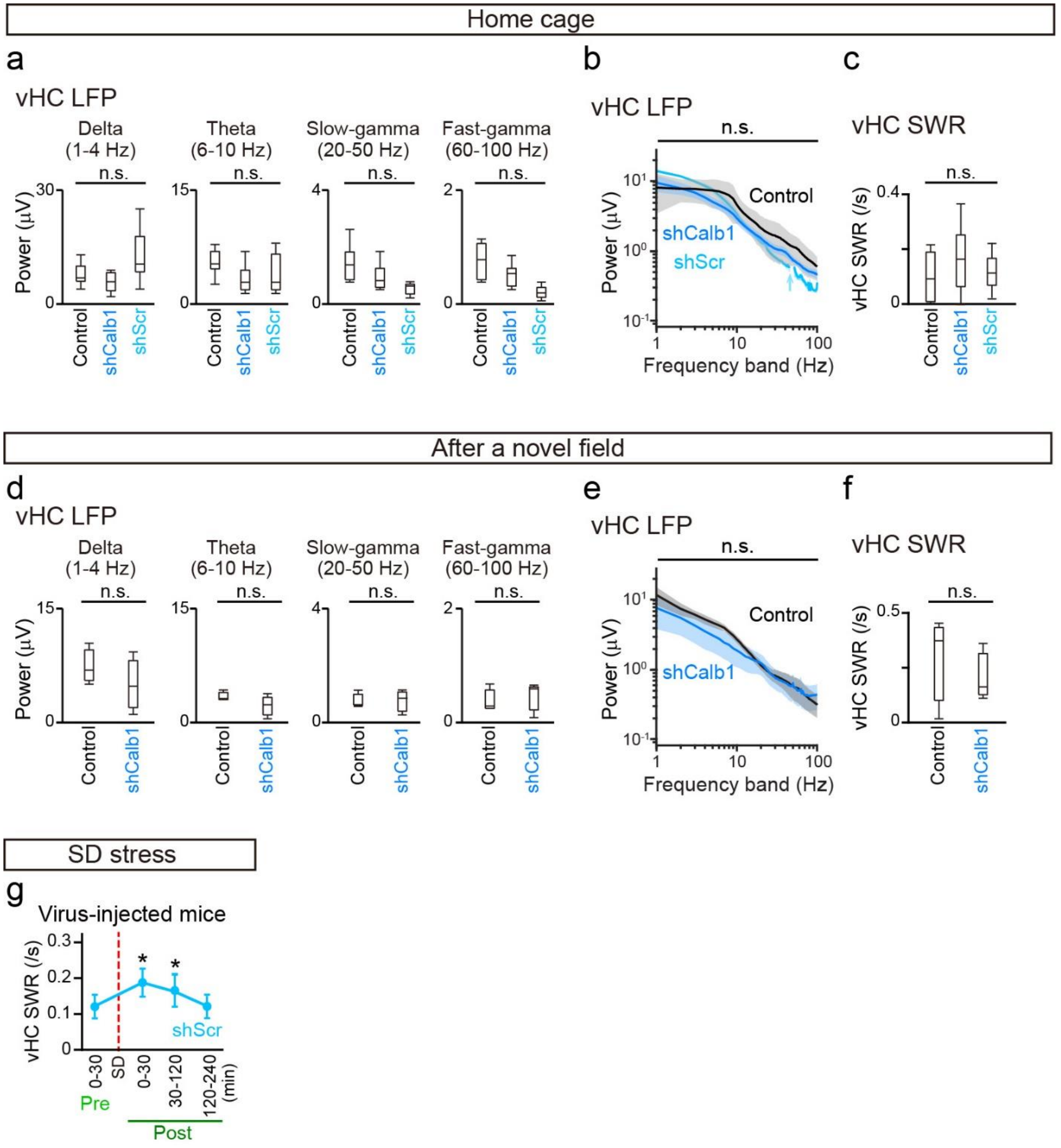

Fig. S2: The effects of Calb1 knockdown and its control on LFP signals in the vHC.

a Comparison of vHC LFP power in the delta (1–4 Hz), theta (6–10 Hz), slow-gamma (20–50 Hz), and fast-gamma (60–100 Hz) bands in a home cage among control (no virus injection; n = 23 mice), AAV-shCalb1-injected, and AAV-shScr-injected mice (n = 11 and 5 mice; $P > 0.05$, two-sided Mann-Whitney U test followed by Bonferroni correction at all bands). Box plots show center line as median, box limits as upper and lower quartiles, whiskers as minimum to maximum values. **b** Comparison of vHC LFP power spectrograms in the home cage, corresponding with a, among the control (black), AAV-shCalb1-injected (cyan), and AAV-shScr-injected (gray) mice during a rest period without SD. Data are presented as the mean ± SEM. The thin cyan arrow indicates a

50-Hz noise signal, excluded by an anlaysis. No significant differences in LFP power were observed at all bands ($q < 0.05$, FDR corrected, two-sided paired *t*-test). **c** Comparison of the rates of vHC SWRs in the home cage (n = 23, 11, and 5 mice) ($P > 0.05$, two-sided Mann–Whitney U test). Box plots show center line as median, box limits as upper and lower quartiles, whiskers as minimum to maximum values. **d** Comparison of vHC LFP power after experiencing a novel open field for 10 min between control (n = 5 mice) and AAV-shCalb1-injected mice (n = 4 mice) ($P > 0.05$, two-sided Mann-Whitney *U* test at all frequency band). Box plots show center line as median, box limits as upper and lower quartiles, whiskers as minimum to maximum values. **e** Comparison of vHC LFP power spectrograms, corresponding with d, between control (black) and AAV-shCalb1-injected (cyan) mice. Data are presented as the mean ± SEM. No significant differences in LFP power were observed in all bands ($q < 0.05$, FDR corrected, two-sided paired *t*-test). **f** Comparison of the rates of vHC SWRs after experiencing a novel open field (n = 5 and 4 mice) ($P > 0.05$, two-sided Mann–Whitney U test). Box plots show center line as median, box limits as upper and lower quartiles, whiskers as minimum to maximum values. **g** Changes in the rates of ventral hippocampal SWRs in mice injected with AAV-shScr into the vHC (n = 5 mice). *P = 0.015 versus the 0–30-min prestress period, bootstrap analysis (two-sided). The data are the mean ± SEM.

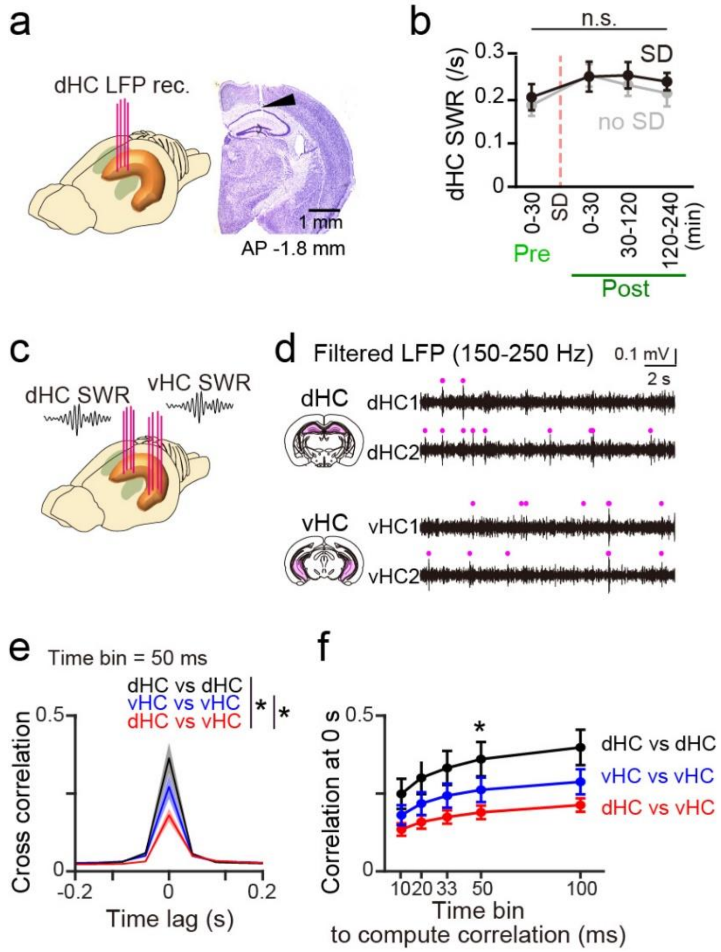

Fig. S3: The dorsal hippocampus showed no pronounced changes in SWR rates in response to SD stress.

a (Left) LFP recordings were obtained from the dHC. (Right) Histological confirmation of a recording site in the dHC CA1 cell layer in a cresyl-stained section. The arrowhead indicates the track of an electrode. The same results were obtained from 9 mice with electrodes implanted in the dHC. **b** Changes in the frequency of the dHC SWRs in SD mice (black, n = 5 mice). For comparison, the same recordings were performed from the mice with non-SD (gray, n = 4 mice). $P > 0.05$, two-sided Mann-Whitney U test versus the 0–30-min prestress period followed by Bonferroni correction. Data are the mean ± SEM. **c** LFP signals were simultaneously recorded from the dHC and vHC. **d** Simultaneously recorded LFP signals (filtered at 150–250 Hz) from two electrodes each from the dHC and vHC. Magenta dots above represent SWRs. **e** Cross-correlograms of SWRs between a pair of electrodes in different regions (dHC vs dHC, n = 11 electrode pairs from 5 mice; dHC vs vHC, n = 39 electrode pairs from 5 mice; vHC vs vHC, n = 12 electrode pairs from 5 mice). With a bin window of 50 ms, for each electrode, a binary sequence was created, consisting of 1 or 0 depending on whether the electrode showed SWRs or not in individual bins. For a given electrode pair, a correlation coefficient was computed from their binary sequences. Electrode pairs located within the same portions of the hippocampus showed significantly higher positive correlations at a time lag of 0 ms, compared with different portions

of the hippocampus (dHC vs dHC versus dHC vs vHC: *Z* = 3.44, **P* = 0.0017; dHC vs dHC versus vHC vs vHC: *Z* = 1.48, *P* = 0.42; dHC vs vHC versus vHC vs vHC: *Z* = 2.54, **P* = 0.033, two-sided Mann-Whitney *U* test versus SD followed by Bonferroni correction), suggesting more independent occurrence of SWRs between the dHC and vHC. **f** Same as e, correlation coefficients at a time lag of 0 ms were computed with a bin window of 10, 20, 33, 50, and 100 ms. For all bin windows, the correlations between the dHC vs dHC and between the vHC vs vHC were higher than those between dHC and vHC, while they were not significantly different, except for the bin window of 50 ms (dHC vs dHC versus dHC vs vHC: *Z* = 3.44, **P* = 0.0017; dHC vs dHC versus vHC vs vHC: *Z* = 1.48, *P* = 0.42; dHC vs vHC versus vHC vs vHC: *Z* = 2.54, **P* = 0.033, two-sided Mann-Whitney *U* test versus SD followed by Bonferroni correction).

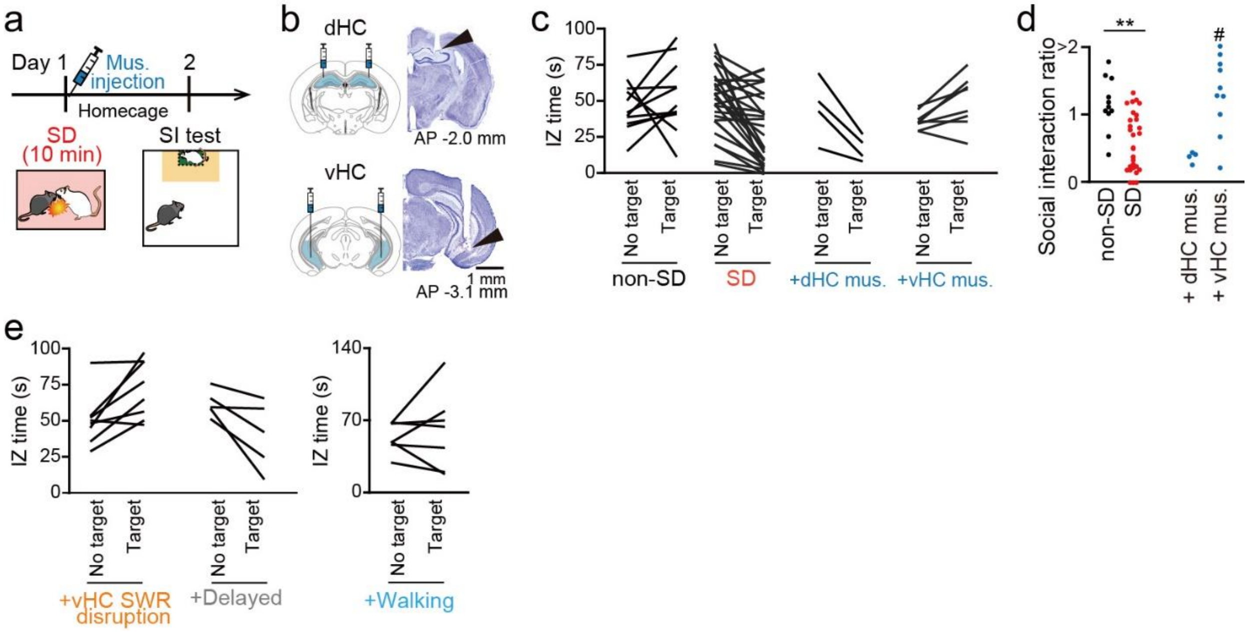

Fig. S4: The effects of 1-day SD stress in mice implanted with an experimental device.

a Experimental timeline for 1-day SD. To determine whether SD-induced social interaction deficits are mediated by a memory reactivation process after SD stress in the hippocampus, the dorsal HC (dHC) or the vHC was inhibited by local infusion of muscimol. **b** Histological verification of cannulae for bilateral muscimol injection into the dHC and vHC. **c** Individual occupancy time periods in the interaction zone (IZ) for each non-SD, SD mouse, and SD mouse with muscimol injection into the dHC and vHC. Each line represents a mouse. Mice with IZ times greater than 100 s are not shown in this graph. **d** The results from C are converted to SI ratios (n = 11, 31, 4, and 10 mice). Each dot represents one mouse. Data of the non-SD, SD, and vHC muscimol groups are similar to Fig. 4g. Non-SD versus SD, $Z = 3.23$, $^{**}P = 0.0036$; SD versus vHC muscimol, $Z = 3.26$, $^{\#}P = 0.0033$, two-sided Mann-Whitney U test versus SD followed by Bonferroni correction. **e** Individual occupancy time periods in the IZ for each mouse presented in Fig 5h and 5k.

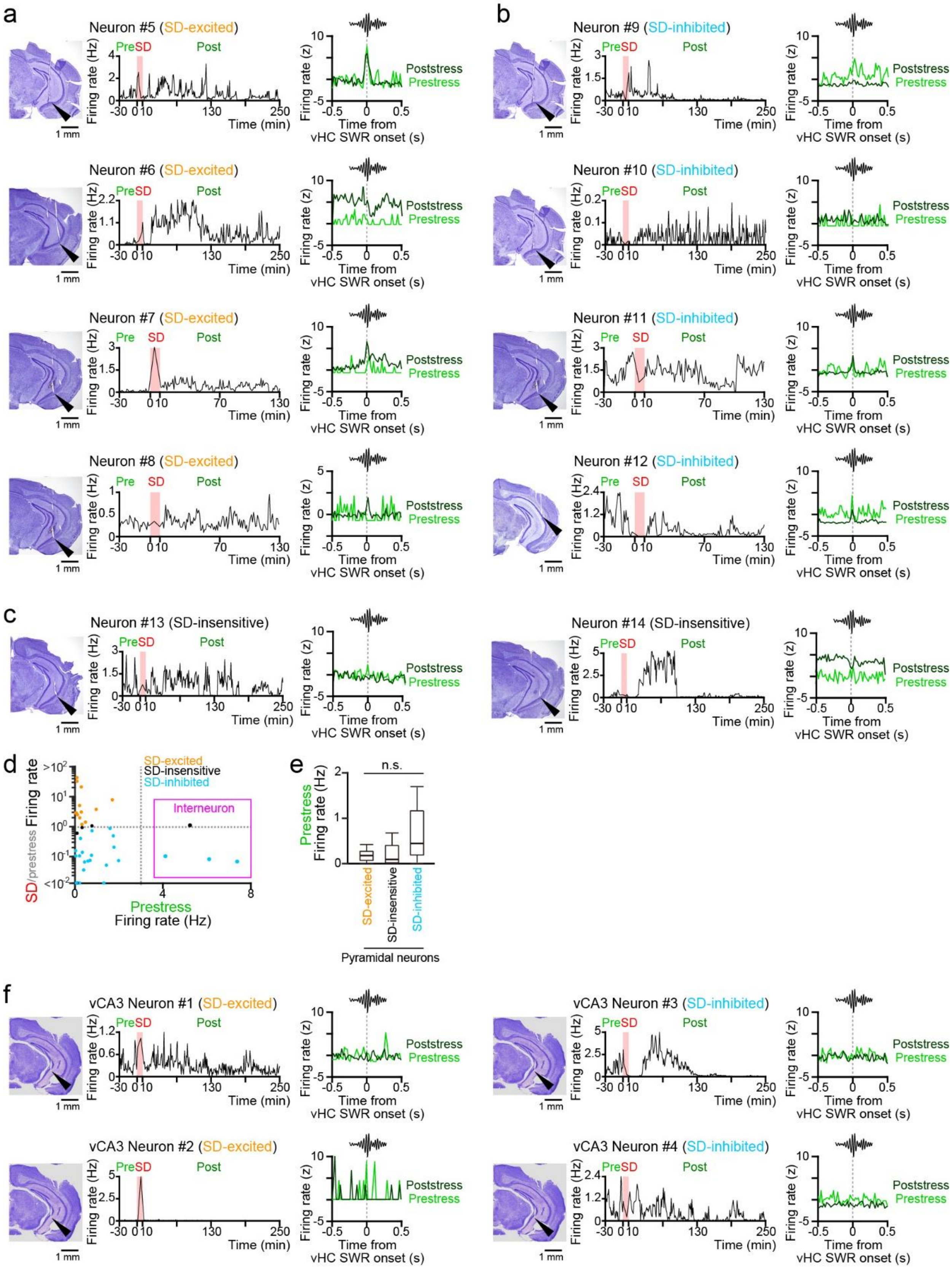

a
Neuron #5 (SD-excited)
Neuron #6 (SD-excited)
Neuron #7 (SD-excited)
Neuron #8 (SD-excited)
b
Neuron #9 (SD-inhibited)
Neuron #10 (SD-inhibited)
Neuron #11 (SD-inhibited)
Neuron #12 (SD-inhibited)
c
Neuron #13 (SD-insensitive)
Neuron #14 (SD-insensitive)
Pre SD Post
Firing rate (Hz)
Time (min)
Firing rate (z)
Poststress
Prestress
Time from vHC SWR onset (s)
1 mm
d
SD-excited
SD-insensitive
SD-inhibited
Interneuron
SD/prestress Firing rate
Prestress Firing rate (Hz)
e
n.s.
Prestress Firing rate (Hz)
SD-excited
SD-insensitive
SD-inhibited
Pyramidal neurons
f
vCA3 Neuron #1 (SD-excited)
vCA3 Neuron #2 (SD-excited)
vCA3 Neuron #3 (SD-inhibited)
vCA3 Neuron #4 (SD-inhibited)

Fig. S5: SWR-induced reactivation of vHC neurons encoding an SD experience. **a** Four representative vHC CA1 SD-excited neurons. (From left to right) Histological confirmation of a recording site, changes in the instantaneous firing rates (bin = 1 and 10 min in the prestress or poststress and SD periods, respectively), and vHC SWR-triggered spike rate changes in the prestress and poststress periods. **b** Same as a but for four vHC CA1 SD-inhibited neurons. **c** Same as a but for two vHC CA1 SD-insensitive neurons. **d** Relationship between the firing rate in the prestress period and the ratio of the firing rate in the SD period to that in the prestress period. Each dot represents a neuron, labeled in different colors (n = 15, 5, 20, and 4 neurons). The magenta region represents 4 putative interneurons, whereas the other cells were putative pyramidal neurons. **e** Boxplots of firing rates in the prestress period for individual cell types of putative pyramidal neurons (n = 15, 5, and 20 neurons; SD-excited versus SD-insensitive, Z = 0.26, P > 0.99; SD-excited versus SD-inhibited, Z = 1.92, P = 0.17; SD- insensitive versus SD-inhibited, Z = 1.66, P = 0.29, two-sided Mann–Whitney U test follow by Bonferroni correction). Box plots show center line as median, box limits as upper and lower quartiles, whiskers as minimum to maximum values. **f** Same as a and b but for two vHC CA3 SD-excited and two vHC CA3 SD-inhibited neurons.

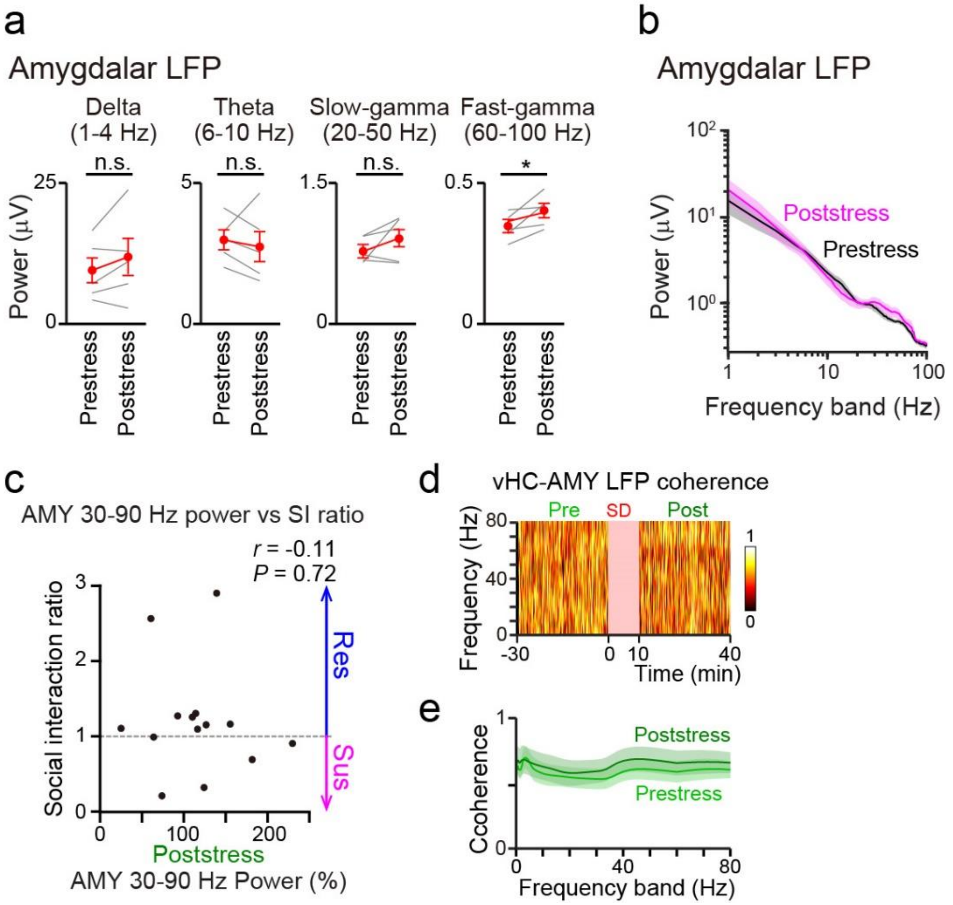

Fig. S6: Analyses of amygdalar LFP signals.

a Comparison of amygdalar LFP power in the delta (1–4 Hz), theta (6–10 Hz), slow-gamma (20–50 Hz), and fast-gamma (60–100 Hz) bands between the prestress and poststress periods (n = 5 mice, delta: t_4 = 1.39, P = 0.24; theta: t_4 = 0.56, P = 0.60; slow-gamma: t_4 = 1.31, P = 0.26; fast-gamma: t_4 = 2.84, *P = 0.047, paired *t test*, two-sided paired *t test*). The data are the mean ± SEM. **b** Comparison of amygdalar LFP power spectrograms between the prestress (black) and poststress (magenta) periods. Data are presented as the mean ± SEM. **c** Relationship between changes in amygdalar 30–90 Hz power in the poststress period and subsequent SI ratios (Pearson's correlation coefficient = –0.11, P = 0.72, two-sided). Each dot represents each mouse (n = 5 uninjected SD mice, 5 AAV-shCalb1-injected mice, and 4 mice injected with muscimol into the vHC). **d** Spectrum of vHC-AMY LFP coherence in a mouse before and after SD stress. The SD period was removed from the analysis. **e** No significant differences in vHC-AMY LFP coherence in each frequency band between the 30-min prestress and poststress periods (n = 5 mice). P > 0.05 at all frequency bands, paired *t test*. The data are the mean ± SEM.

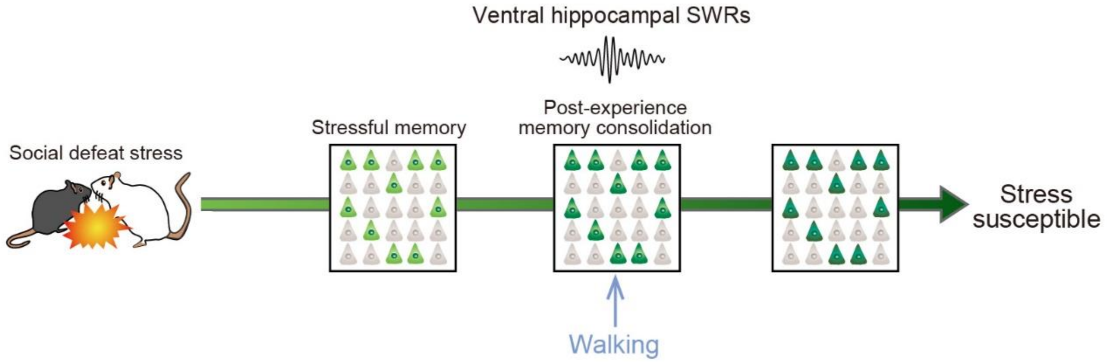

Fig. S7: A schematic illustration summarizing this study.

(From left to right) A black mouse experiences social defeat stress from a white mouse. Hippocampal neurons encoding the stressful event are activated. The strength of memory reactivation by ventral hippocampal SWRs is related to subsequent stress susceptibility
