# Supplementary material for: Hippocampal sharp wave ripples underlie stress susceptibility in male mice
Source: Nat Commun. 2023 Apr 20;14:2105. doi: 10.1038/s41467-023-37736-x (PMC10119298; doi:10.1038/s41467-023-37736-x)
Supplement: Supplementary file 2 — Reporting Summary [file 41467_2023_37736_MOESM2_ESM.pdf]

nature portfolio

Corresponding author(s): Takuya Sasaki
Last updated by author(s): Feb 14, 2023

Reporting Summary

Nature Portfolio wishes to improve the reproducibility of the work that we publish. This form provides structure for consistency and transparency in reporting. For further information on Nature Portfolio policies, see our Editorial Policies and the Editorial Policy Checklist.

Statistics

For all statistical analyses, confirm that the following items are present in the figure legend, table legend, main text, or Methods section.

n/a	Confirmed	
☐	☒	The exact sample size (*n*) for each experimental group/condition, given as a discrete number and unit of measurement
☒	☐	A statement on whether measurements were taken from distinct samples or whether the same sample was measured repeatedly
☐	☒	The statistical test(s) used AND whether they are one- or two-sided<br>*Only common tests should be described solely by name; describe more complex techniques in the Methods section.*
☒	☐	A description of all covariates tested
☐	☒	A description of any assumptions or corrections, such as tests of normality and adjustment for multiple comparisons
☐	☒	A full description of the statistical parameters including central tendency (e.g. means) or other basic estimates (e.g. regression coefficient) AND variation (e.g. standard deviation) or associated estimates of uncertainty (e.g. confidence intervals)
☐	☒	For null hypothesis testing, the test statistic (e.g. *F*, *t*, *r*) with confidence intervals, effect sizes, degrees of freedom and *P* value noted<br>*Give P values as exact values whenever suitable.*
☒	☐	For Bayesian analysis, information on the choice of priors and Markov chain Monte Carlo settings
☒	☐	For hierarchical and complex designs, identification of the appropriate level for tests and full reporting of outcomes
☒	☐	Estimates of effect sizes (e.g. Cohen's *d*, Pearson's *r*), indicating how they were calculated

Our web collection on statistics for biologists contains articles on many of the points above.

Software and code

Policy information about availability of computer code

Data collection	The collected tissue sample was immediately frozen in liquid nitrogen. Total RNA was prepared from each of the mice using a RNeasy Plus Mini Kit (Qiagen Inc.). A Cereplex Direct data acquisition system (Blackrock Microsystems) was used for electrophysiological data collection. CMOS camera (MCM4350, Gazo) attached on the ceiling was used for recording animal's behavior.
Data analysis	RNAs were applied to microarray analysis performed by Affymetrix GeneChip Mouse Clariom S arrays (Kurabo Industries Ltd., Osaka, Japan). The prepared microarrays were preprocessed with Transcriptome Viewer (Kurabo Industries Ltd., Osaka, Japan). For All electrophysiological data analysis, Matlab2019b was used. For spike sorting, the graphical cluster-cutting software MClust4.3.02 was used. Animal's trajectories in images were manually extracted by Image J1.45.

For manuscripts utilizing custom algorithms or software that are central to the research but not yet described in published literature, software must be made available to editors and reviewers. We strongly encourage code deposition in a community repository (e.g. GitHub). See the Nature Portfolio guidelines for submitting code & software for further information.

Data

Policy information about availability of data

All manuscripts must include a data availability statement. This statement should provide the following information, where applicable:

- Accession codes, unique identifiers, or web links for publicly available datasets
- A description of any restrictions on data availability
- For clinical datasets or third party data, please ensure that the statement adheres to our policy

All data needed to evaluate the conclusions are described in the manuscript and/or Supplementary Materials. The original gene expression data are deposited in DDBJ Genomic Expression Archive (GEA) under accession number E-GEAD-490. Original physiological datasets are provided on Mendeley Data (https://data.mendeley.com/datasets/7jdtbdx2gp/1). Source data are provided with this paper.

Human research participants

Policy information about studies involving human research participants and Sex and Gender in Research.

Reporting on sex and gender	N/A
Population characteristics	N/A
Recruitment	N/A
Ethics oversight	N/A

Note that full information on the approval of the study protocol must also be provided in the manuscript.

Field-specific reporting

Please select the one below that is the best fit for your research. If you are not sure, read the appropriate sections before making your selection.

☒ Life sciences ☐ Behavioural & social sciences ☐ Ecological, evolutionary & environmental sciences

For a reference copy of the document with all sections, see nature.com/documents/nr-reporting-summary-flat.pdf

Life sciences study design

All studies must disclose on these points even when the disclosure is negative.

Sample size	No prior sample-size calculation was performed as no previous datasets could be used to estimate differences in RNA expression levels and the properties of sharp wave ripples. Sample size was determined to be adequate based on the magnitude and consistency of measureable differences among groups.
Data exclusions	For behavioral and electrophysiological tests, no exlcusions of mice, except ones that were dead after surgery, were performed. For microarray data analysis, raw signals were transformed to the log2 scale and then normalized. In cases where the probes for a given gene yielded a p-value (detection p-value) greater than 0.05, the gene was excluded from further analysis.
Replication	For behavioral and electrophysiological tests, all attempts at replication were successful. Independent behavioral and electrophysiological experiments were repeated three to four times to get the similar results.
Randomization	For all experiments, mice were randomly assigned to experimental groups by sampling across different litters.
Blinding	All the data and influences in this study were analyzed and generated completely independently. Hence blinding was not applicable as there was no bias present.

Reporting for specific materials, systems and methods

We require information from authors about some types of materials, experimental systems and methods used in many studies. Here, indicate whether each material, system or method listed is relevant to your study. If you are not sure if a list item applies to your research, read the appropriate section before selecting a response.

Materials & experimental systems

n/a	Involved in the study	
☐	☒	Antibodies
☒	☐	Eukaryotic cell lines
☒	☐	Palaeontology and archaeology
☐	☒	Animals and other organisms
☒	☐	Clinical data
☒	☐	Dual use research of concern

Methods

n/a	Involved in the study	
☒	☐	ChIP-seq
☒	☐	Flow cytometry
☒	☐	MRI-based neuroimaging

Antibodies

Antibodies used

Primary antibodies: rabbit anti-NeuN antibody (1:2000, ab177487, Abcam), goat anti-Calbindin antibody (1:400; Calbindin-Go-Af1040, Frontier Institute Co., Ltd.)
Fluorophore-conjugated secondary antibodies: anti-rabbit IgG antibody Alexa 647 (1:1000; Thermo Fisher Scientific,), anti-Goat IgG antibody Alexa 488 (1:1000; Thermo Fisher Scientific)

Validation

primary rabbit anti-NeuN antibody (https://www.abcam.com/neun-antibody-epr12763-neuronal-marker-ab177487.html)
primary goat anti-Calbindin antibody (https://nittobo-nmd.co.jp/pdf/reagents/Calbindin.pdf)

Animals and other research organisms

Policy information about studies involving animals; ARRIVE guidelines recommended for reporting animal research, and Sex and Gender in Research

Laboratory animals

Male C57BL/6J mice (10–15 weeks old) with preoperative weights of 22–35 g were used for all data sampling. In addition, male CD-1 mice (13-20 weeks old) with weights of 40–50 g were used as affressor mice that imposed social defeat stress. They were individually housed and maintained on a 12-h light/12-h dark schedule under housing conditions at 23 ± 1°C with relative humidity of 50 ± 5% with lights off at 8:00 AM. All mice were purchased from SLC (Shizuoka, Japan).

Wild animals

No wild animals were used.

Reporting on sex

Only male mice were used in this study because the experimental paradigms of social defeat stress employed in this study has been established for male mice. Importantly, the stress models using male aggressor mice are only applicable to male mice.

Field-collected samples

No field-collceted samples were used.

Ethics oversight

All experiments were performed with the approval of the animal experimental ethics committee at the University of Tokyo (approval number: P29-14) and the committee on animal experiments at Tohoku University (approval number: 2022 PhA-004) and in accordance with the NIH guidelines for the care and use of animals.

Note that full information on the approval of the study protocol must also be provided in the manuscript.
